# Adaptations of mitochondrial, autophagy and nutrient sensing pathways in the liver from long-lived mice overexpressing CYB5R3 are sex-dependent and involve inter-organ responses

**DOI:** 10.1007/s11357-025-01761-z

**Published:** 2025-06-28

**Authors:** Luz Marina Sánchez-Mendoza, José A. González-Reyes, Sandra Rodríguez-López, Cristina García-Caballero, Juan Antonio Moreno, Rafael de Cabo, M. Isabel Burón, José M. Villalba

**Affiliations:** 1 Departamento de Biología Celular, Fisiología e Inmunología, Universidad de Córdoba, Campus de Excelencia Internacional Agroalimentario, ceiA3, 14014 Córdoba, Spain; 2 Maimonides Biomedical Research Institute of Cordoba (IMIBIC), Hospital Universitario Reina Sofía, Córdoba, Spain; 3 Experimental Gerontology Section, Translational Gerontology Branch, National Institute on Aging, National Institutes of Health, Baltimore, MD 21224 USA

**Keywords:** CYB5R3, Liver, Mitochondria, Lipid metabolism, Sexual dimorphism

## Abstract

Cytochrome *b*_5_ reductase 3 (CYB5R3) overexpression mimics several metabolic benefits of calorie restriction, with sex- and tissue-specific effects. This study aimed to investigate how CYB5R3 overexpression impacts hepatic metabolism in young mice, focusing on mitochondrial biogenesis, lipid metabolism, autophagy and nutrient sensing pathways thus establishing a baseline that allows for subsequent comparisons with older animals. The accrual of CYB5R3 polypeptide exhibited marked sexual dimorphism as it was increased by transgenesis only in females with predominant microsomal targeting but mainly located in the mitochondria in males. Nevertheless, key metabolic markers, including TFAM (biogenesis), p62 and LC3 (autophagy), AKT and mTOR (nutrient sensing), SIRT1/SIRT3 (enzymatic regulation and gene expression) and ACC1/ACAA2 (lipid metabolism), were significantly altered in transgenic males. Wild-type males exhibited higher levels of mitochondrial complexes III and IV than females, and these differences were attenuated by transgenesis. TFAM was increased in transgenic mice of both sexes, indicative of enhanced mitochondrial biogenesis. Changes of lipid metabolism markers indicated reduced hepatic lipid accumulation in females while males showed changes in ACC1 and ACAA2, affecting lipid storage and oxidation. Autophagy markers (p62, LC3I/II) were altered in males, whereas mitophagy markers (PINK1, PARKIN) were upregulated in females, suggesting efficient mitochondrial turnover. In conclusion, CYB5R3 expression is regulated by post-transcriptional and post-translational mechanisms and induces sex-dependent hepatic metabolic adaptations. While females exhibit increased CYB5R3 protein and associated mitochondrial improvements, males respond with distinct metabolic reprogramming despite unchanged CYB5R3 levels, underscoring the relevance of sexual dimorphism and systemic regulatory mechanisms in the response to longevity-promoting interventions.

**Supplementary Information:**

The online version contains supplementary material available at 10.1007/s11357-025-01761-z.

## Introduction

The flavoenzyme NADH-cytochrome *b*_5_ reductase-3 (CYB5R3, EC 1.6.2.2) is expressed either ubiquitously as a membrane protein attached to the cytosolic side of the outer mitochondrial membrane, the endoplasmic reticulum and the plasma membrane, or as a soluble isoenzyme exclusive of the erythroid lineage [1]. The membrane-bound isoform transports electrons from NADH to a variety of acceptors, with several heme-containing proteins being the best characterized ones, as the two cytochrome *b*_5_ (CYB5) isoforms (CYB5A at the endoplasmic reticulum and CYB5B at the outer mitochondrial membrane), soluble guanylate cyclase (sGC) and globins as cytoglobin and myoglobin [1]. This isoform can also reduce non-heme acceptors as the lipophilic antioxidant and electron carrier coenzyme Q (CoQ) [2] which, in its reduced ubiquinol form, recycles plasma membrane α-tocopherol and extracellular ascorbate [3], and modulates NOX4 activity at the outer mitochondrial membrane by regulating superoxide and peroxide levels [4]. The erythroid-specific soluble CYB5R3 isoform reduces methemoglobin to hemoglobin and facilitates oxygen transport in erythrocytes [5].

Our previous observation that CYB5R3 abundance and activity can be upregulated in some tissues of mice, rats and cells subjected to calorie restriction (CR) conditions [6–9] prompted us to develop a transgenic mouse model of CYB5R3 overexpression (TG-mice). TG-mice were found to live longer than their WT counterparts and to exhibit some of the metabolic improvements that are also shown with CR and other interventions promoting a healthier phenotype with aging, as decreased oxidative damage, better glucose homeostasis, less inflammation and enhanced protection against induced cancer [10].

Further characterization of this model has allowed us to demonstrate that metabolic adaptations elicited by CYB5R3 overexpression are substantially sex- and tissue-specific. At the organismal level, TG males primarily rely on carbohydrate metabolism and exhibit increased fat content [10], whereas TG females are characterized by increased fatty acid oxidation and accumulate less fat [8]. In skeletal muscle, CYB5R3 overexpression upregulated markers consistent with enhanced mitochondrial biogenesis and function, and increased mitochondrial abundance, producing its potentially beneficial actions mostly in females [11]. In epithelial cells from kidney distal tubules, CYB5R3 overexpression mitigated age-related changes in mitochondrial size and abundance, as well as in mitochondria-endoplasmic reticulum contact sites (MERCS), producing its effects mostly in females as well [12]. In the heart, however, CYB5R3 overexpression upregulated markers consistent with enhanced mitochondrial function mainly in males [13], illustrating the complexity of the CYB5R3-overexpressing transgenic model.

Since liver accounts for a substantial part of resting energy expenditure [14], we hypothesized that CYB5R3 overexpression could affect differentially key hepatic metabolic pathways in a sex-dependent way. The present study was designed to investigate in the liver how sex and CYB5R3 overexpression influence key markers associated with well-established pathways that play a critical role in shaping the aging process, such as mitochondrial respiration and fatty acid utilization, mitochondrial dynamics and biogenesis, general autophagy and mitophagy, and nutrient sensing [15]. Ultrastructure features of liver mitochondria and lipid droplets (LDs) were also studied by transmission electron microscopy.

Our results have indicated that the accrual of hepatic CYB5R3 exhibits sexual dimorphism and is subjected to post-transcriptional and post-translational regulation mechanisms. Moreover, overexpression of CYB5R3 gene increased CYB5R3 polypeptide in the liver from females but not males and produced distinct sex-dependent biochemical signatures. Despite the lack of CYB5R3 increase in the liver from TG males, key metabolic markers as TFAM (mitochondrial biogenesis), p62, LC3 I and II (autophagy), PARKIN and PINK1 (mitophagy), AKT and mTOR (anabolic/catabolic pathways), SIRT1 and SIRT3 and protein pan-acetylation (regulation of enzymatic activity and gene expression) and ACC1 and ACAA2 (lipogenesis/lipolysis) were still significantly altered in TG compared with WT males. Our observations support that non-cell autonomous mechanisms likely related with inter-organ communication contribute to determine the sex-dependent and tissue-specific outcome of CYB5R3 overexpression in mice.

## Materials and methods

### Establishment of experimental groups

CYB5R3-overexpressing transgenic mice (TG mice) were generated as previously described (see [10] and Supplemental Methods). Mice were maintained from weaning on 12-h light/dark cycle at 22 °C with a free access to water and a standard chow at the Service of Experimentation Animals (SAEX) of the University of Córdoba. All determinations described in this paper were carried out with 3-month-old mice. Four experimental groups (*n* = 5–7 mice per group) were established for this study: WT females (WTF), WT males (WTM), TG females (TGF) and TG males (TGM).

### Procurement of tissue samples

Mice were anesthetized with isoflurane (at 4% for induction and at 1.5% for maintenance of anaesthesia) with an O_2_ flow of 0.8–1 l/min, exsanguinated by cardiac puncture and euthanized by cervical dislocation. Then, the liver was rapidly excised and snap-frozen in liquid nitrogen in a buffered medium containing 10% DMSO as cryoprotectant. Tissue samples were stored at −  80 °C until analysis. Small pieces from the left lateral lobe were also obtained and immediately processed for electron microscopy as described in a separate section (see below). Procedures with experimentation animals were approved by the bioethics committee of the University of Córdoba and authorized by the *Consejería de Agricultura, Pesca y Desarrollo Rural*, *Junta de Andalucía, Spain* (authorization code: 06/06/2019/098).

### Preparation of whole hepatic extracts

The liver was homogenized for 30 s in radioimmunoprecipitation assay (RIPA) buffer (50 mM Tris–HCl pH 8, 150 mM NaCl, 0.5% deoxycholate, 0.1% SDS, 1% Triton X-100, 1 mM DTT, 1 mM phenylmethylsulphonyl fluoride (PMSF), 10 μg/mL each of chymostatin, leupeptin, antipain, and pepstatin A (CLAP), and phosphatase inhibitor cocktails 2 and 3 (Sigma-Aldrich) diluted at 1:100). Tissue homogenization was carried out using a mechanical tissue disrupter (Ultra-Turrax T25, IKA, Staufen, Germany) for 30 s, or a MM400 steel ball mill (Retsch, Germany) for 2 min at 25 s^−1^. The homogenates were centrifuged at 10,000 × *g* for 15 min at 4 °C to separate whole protein extracts in the supernatants, which were then transferred to clean tubes and stored frozen at −  80 °C until use.

### Subcellular fractionation

Liver samples were dispersed with an electric tissue disrupter (Ultra-Turrax T25, IKA, Staufen, Germany) for 30 s at 4 °C in a homogenization buffer composed of 20 mM Tris–HCl pH 7.6, 40 mM KCl, 0.2 M sucrose, 1 mM PMSF, 10 mM EDTA, 1 mM DTT, 20 μg/μL CLAP and phosphatase inhibitor cocktails 2 and 3 (Sigma-Aldrich) diluted at 1/100. Total homogenates were centrifuged at 420 × *g* for 10 min to sediment cell debris and nuclei that were discarded. The supernatants derived from this step were collected and centrifuged again at 6700 × *g* for 10 min to sediment a heavy (mitochondria-enriched) membrane fraction that was resuspended in 100 μl of isolation buffer and stored at −  80 °C for further analysis. Supernatants derived from this last centrifugation step were transferred to clean ultracentrifuge tubes and spun again at 100,000 × *g* for 30 min to sediment a fraction of light membranes (enriched in microsomes and plasma membrane-derived vesicles) that was resuspended and stored frozen in the same way. Total amount of protein present in RIPA extracts and in subcellular fractions was estimated using the Bradford dye-binding method as modified by Stoscheck [16].

### Electrophoresis and Western blot immunodetection

Electrophoresis and electro-transference to nitrocellulose sheets were carried out as described previously [17]. Membranes were then probed against the primary antibodies listed in Table 1. The corresponding species-specific secondary antibodies coupled to horseradish peroxidase were used to reveal binding sites by enhanced chemiluminescence (ClarityTM Western ECL Blotting Substrates Kit, Bio-Rad). Chemiluminescence signals were recorded using a ChemiDoc Imaging System (Bio-Rad), and the densitometric quantification of immunostained bands was carried out with Image LabTM Software (Bio-Rad). Data were normalized to the overall image density of the corresponding lane stained with Ponceau S. All samples depicted in each figure were run on the same gel.

**Table 1:** Primary antibodies used in this study. The table shows the concentrations and the commercial references of each antibody. (*SC* Santa Cruz Antibodies)

Primary antibodies	Dilution	Reference	Primary antibodies	Dilution	Reference
**CYB5R3**	1:1000	Proteintech 10,894–1-AP	**DRP1**	1:500	SC-32898
**PINK1**	1:1000	SC-33796	**FIS1**	1:500	SC-98900
**PARKIN**	1:100	Cell Signaling 2132	**VDAC**	1:1000	SC-98708
**NRF1**	1:2000	SC-33771	**TFAM**	1:1000	SC-2358
**MFN1**	1:1000	SC-50330	OxPhos rodent WB antibody cocktail*	1:4000	Life technologies 458,099
**MFN2**	1:500	SC-50331	**ERα**	1:1000	Sigma-Aldrich 06–935
**SIRT1**	1:1000	SC-15404	**SIRT3**	1:1000	SC-99143
**ACC1**	1:1000	Proteintech 21,923–1-AP	**HADH**	1:2000	Proteintech 19,828–1-AP
**pACC1**	1:1000	Proteintech 29,119–1-AP	**ACAA2**	1:1000	Sigma-Aldrich WH0010449M1
**LC3A/B**	1:1000	Cell Signaling 4108	**P62/SQSTM1 Sigma**	1:2000	P0067
**AKT1/2/3 (H-136)**	1:1000	SC-8312	**p-AKT1/2/3 (Ser 473)**	1:500	SC-7985-R
**mTOR (7C10)**	1:1000	Cell Signaling 2983	**p-mTOR (Ser2448)**	1:500	Cell Signaling 2971
**Ac-Lys**	1:1000	Cell Signaling 9441			

^*^OxPhos rodent WB antibody cocktail:

Complex I subunit NDUFB8 (NADH dehydrogenase (ubiquinone) 1 beta subcomplex subunit 8)

Complex II subunit SDHB (Succinate dehydrogenase (ubiquinone) iron-sulfur subunit)

Complex III subunit UQCRC2 (Cytochrome b-c1 complex subunit)

Complex IV subunit MTCO1 (mitochondrially encoded cytochrome c oxidase I)

Complex V subunit ATP5A (Complex V alpha subunit)

### Measurement of estrogen levels in serum

Estrogen levels were determined using an ELISA kit (ab285313, Abcam, UK). Serum was collected and allowed to coagulate for 2 h at room temperature. Subsequently, it was centrifuged at 1000 × *g* for 20 min. The supernatant was collected, and the assay was performed immediately. All experimental steps were strictly conducted according to the manufacturer’s instructions.

### Ultrastructural analysis

A portion of the liver left lateral lobe was cut in small pieces that were fixed with aldehydes, dehydrated in a graded series of ethanols and embedded in epoxy resin. Thin sections were then obtained and stained in uranyl acetate and lead citrate following routine electron microscopy methods (see Supplemental Methods). Stained sections were examined and photographed in a Jeol JEM 1400 electron microscope at the *Servicio Centralizado de Apoyo a la Investigación* (SCAI; University of Córdoba; Spain). Micrographs of hepatocytes were taken at 6000 × and used for planimetric (sectional area, volume and circularity) and stereological analyses of mitochondria and lipid droplets. Stereological analyses were performed to calculate volume density (Vv), defined as the volume occupied by mitochondria per volume unit of the cell (expressed in µm^3^/µm^3^), and numerical profile density (Na), which measures the number of mitochondria per µm^2^ of cell surface. To this purpose, we followed the point counting method of Weibel [18] by superposing the pictures with a simple square lattice with 0.4-µm separation between points. Measurements were performed using ImageJ software (NIH). LD volume was calculated by assimilating these structures to spheres and using the sphere volume formula $Vol=\frac{4}{3}\pi {r}^{3}$. The average radius of the LDs was obtained using Image J software (NIH). About 1000 mitochondria or LDs were scored per experimental group.

### RNA isolation and transcriptomic analyses

Liver samples were homogenized in Trizol (Invitrogen™ 15,596,018) using the Retsch bead mill, applying the disruption for a duration of 2 min at 25 s^−1^, and RNA was extracted as described [19]. Reverse transcription was carried out using the High-Capacity cDNA Reverse Transcription Kit (ThermoFisher) following the manufacturer’s instructions. qPCR reactions were performed in a final volume of 10 μl using a CFX-Connect Real-Time system (Bio-Rad) and master mix (TB Green™ Premix Ex Taq™ (Takara, RR420L)) following the manufacturer’s instructions. The reactions consisted of an initial denaturing step of 30 s at 95 °C followed by 39 cycles of 5 s at 95 °C and 30 s at 60 °C and melting curve of 5 s at 65 °C and 5 s at 95°C. The levels of transcripts were normalized using the geometric average of GADPH. Data were analyzed by 2-ΔΔCt method. Primers sequences are listed in Table 2.

**Table 2:** Sequences of primers used for RT-PCR analyses

	**Forward**	**Reverse**
Cyb5r3	5′-GAAGTCTGTAGGCATGATTG-3′	5′-AACAGGATATCTTTCTCGGAC-3′
GAPDH	5′-TGCACCACCAACTGCTTAGC-3′	5′-GGCATGGACTGTGGTCATGAG-3′

### Statistical analysis

Data were expressed as mean ± SEM. The Kolmogorov–Smirnov test was employed to assess for values normality. Means were compared by two-tailed Student’s *t* test, whereas global effects of sex or genotype were assessed by two-way ANOVA. In case data did not pass the normality test, the nonparametric two-tailed Mann–Whitney test was followed. Significant differences were expressed as follows: *(*p* < 0.05), **(*p* < 0.01), ***(*p* < 0.001) and ****(*p* < 0.0001). All statistical analyses and graphics were performed using GraphPad Prism 8 (GraphPad Software Inc., San Diego, CA).

## Results

### Overexpression of the CYB5R3 gene produces distinct and sex-dependent effects on CYB5R3 mRNA and polypeptide abundance in the liver

**Fig. 1:**
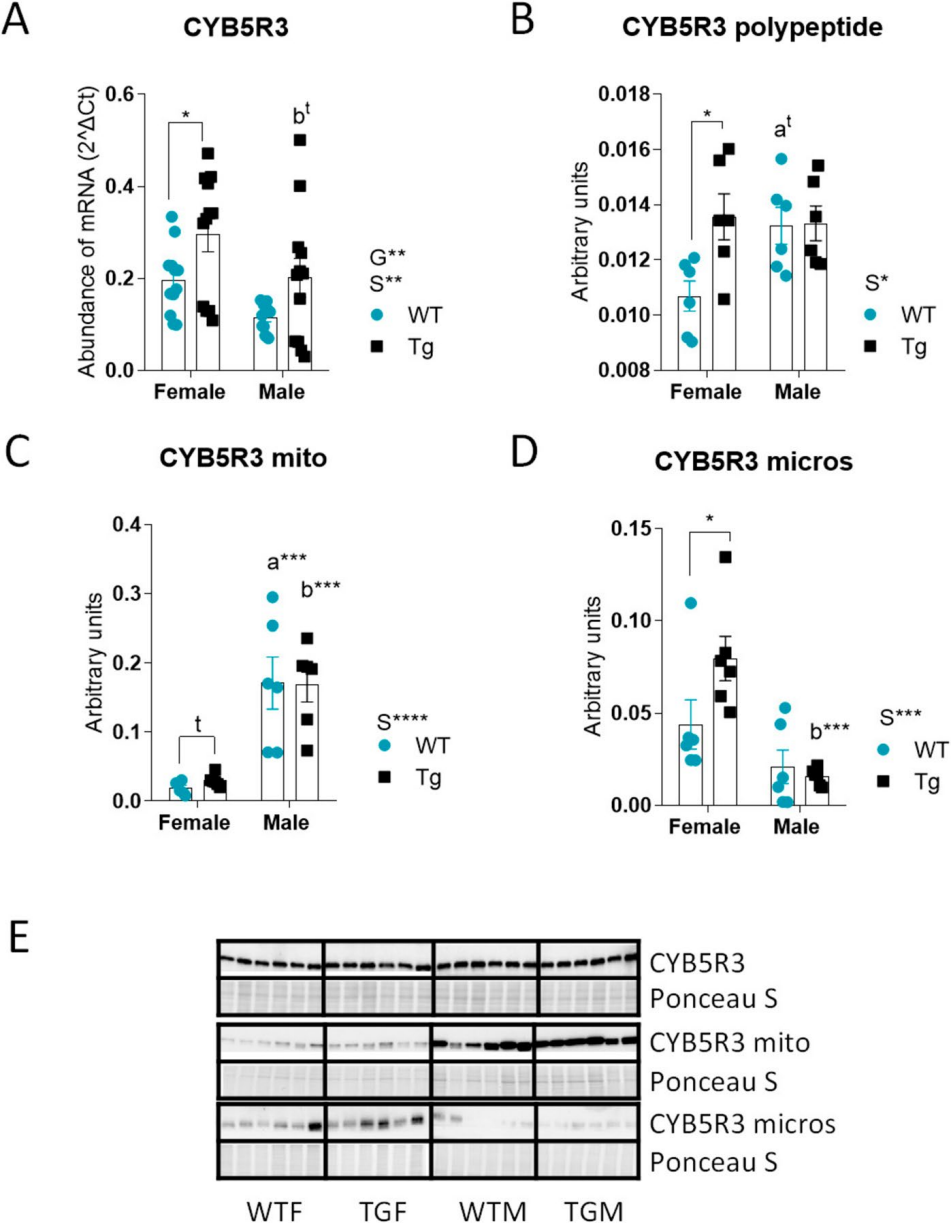
Levels of CYB5R3 transcripts, polypeptide CYB5R3 in whole homogenates, in fractions of mitochondria-enriched and endoplasmic reticulum-enriched (microsomal) in the liver from females and males of WT or TG genotypes. Levels of CYB5R3 transcripts (**A**) were determined by RT-PCR on total RNA, whereas the abundance of CYB5R3 polypeptide in whole homogenates (**B**), in mitochondria-enriched fractions (**C**) and in endoplasmic reticulum-enriched fraction (**D**) were determined by western blot. Western blots used for quantification of protein levels and their corresponding Ponceau S-stained lanes used for normalization of protein loading are shown in panel **E**. Global effects of sex or genotype, as well as the interaction between these factors, were evaluated by two-way ANOVA. Asterisks in the bars denote the level of significance of the differences between genotypes (WT *vs*. TG) for a given sex. Asterisks that are accompanied by “a” (for WT) or “b” (for TG) denote significant differences between females and males for a given genotype. Global effects of sex (regardless of genotype) are represented as “S” with asterisks, while overall effects of genotype (regardless of sex) are represented as “G” with asterisks. The interaction “sex x genotype” is indicated as “I” when appropriate. Data are shown as mean ± SEM of 6 animals per group

We first determined the abundance of CYB5R3 transcripts in the liver from males and females of WT and TG genotypes and found a main effect of sex decreasing CYB5R3 mRNA in males independently of genotype (Fig. 1A). There was also a main effect of genotype increasing CYB5R3 mRNA in TG mice irrespective of sex, with significant differences being observed in TGF *vs*. WTF (Fig. 1A). Next, we studied the abundance of CYB5R3 polypeptide in whole homogenates. Strikingly, despite the observed changes in mRNA levels, CYB5R3 polypeptide was found elevated in males independently of genotype (Fig. 1B). Moreover, when comparisons were made among specific groups, we found a significant elevation of CYB5R3 polypeptide in TGF *vs*. WTF (Fig. 1B), which agreed with the differences in mRNA (see Fig. 1A). In males, however, the abundance of CYB5R3 polypeptide tended to be higher in WTM *vs*. WTF but no differences were encountered when comparing TGM *vs*. WTM (Fig. 1B).

The lack of differences in terms of the abundance of CYB5R3 polypeptide in males was surprising. Since CYB5R3 is targeted mainly to the outer mitochondrial and endoplasmic reticulum membranes [1], we considered the possibility that the accrual of CYB5R3 polypeptide in these membranes could be different in WT and TG mice of both sexes. Thus, we determined CYB5R3 abundance in heavy (mitochondria-enriched) and light (endoplasmic reticulum-enriched) membrane fractions isolated from the homogenates by differential centrifugation. Interestingly, we detected a sex-specific pattern of endogenous CYB5R3 distribution since the mitochondria-associated protein was more abundant in males than in females (Fig. 1C), whereas the opposite was observed for the endoplasmic reticulum-associated protein (Fig. 1D). When we analysed the effect of transgenesis, it was found that the increase of CYB5R3 polypeptide we had encountered in liver homogenates from TGF *vs*. WTF was accounted by similar increases in subcellular membranes. However, no changes were evidenced in males, neither for mitochondrial nor for microsomal membranes (Fig. 1C, D). Figure 1E depicts western blots used for quantification of CYB5R3 in different fractions with their corresponding Ponceau S-stained lanes used for normalization of protein loading.

### Effect of sex and CYB5R3 overexpression on the abundance of VDAC and mitochondrial OXPHOS markers

**Fig. 2:**
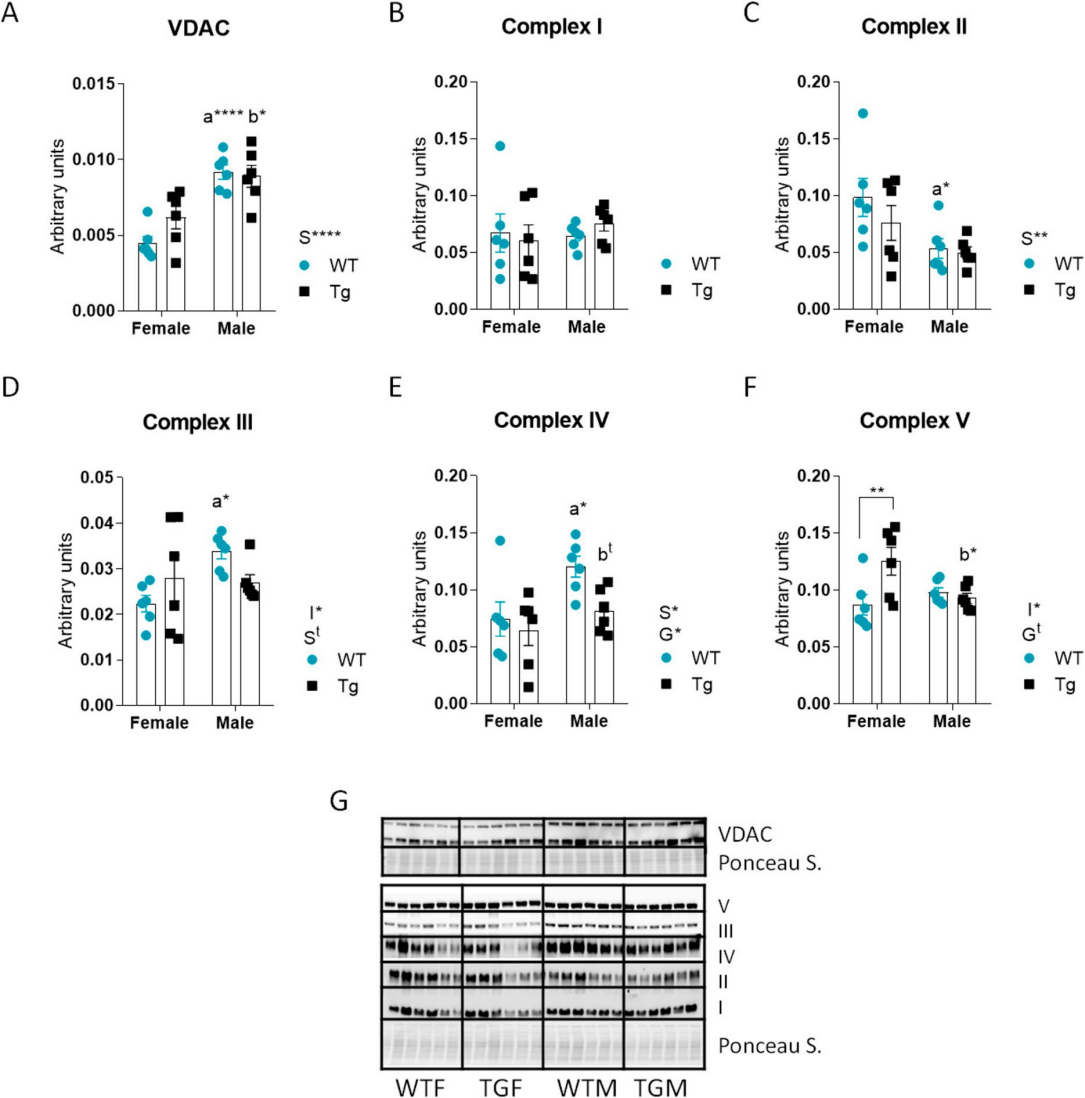
Levels of VDAC and electron transport chain complexes in the liver from females and males of WT or TG genotypes. Panel **A** represents the levels of the mitochondrial mass marker VDAC. Panels **B** to **F** depict the levels of mitochondrial complexes I to V markers, respectively. Western blots used for quantification of protein levels and their corresponding Ponceau S-stained lanes used for normalization of protein loading are shown in panel **G**. Global effects of sex or genotype, as well as the interaction between these factors, were evaluated by two-way ANOVA. Asterisks in the bars denote the level of significance of the differences between genotypes (WT *vs*. TG) for a given sex. Asterisks or “t” (trend) that are accompanied by “a” (for WT) or “b” (for TG) denote significant differences between females and males for a given genotype. Global effects of sex (regardless of genotype) are represented as “S” with asterisks or “t,” while overall effects of genotype (regardless of sex) are represented as “G” with asterisks or “t.” The interaction “sex x genotype” is indicated as “I” when appropriate. Data are shown as mean ± SEM of 6 animals per group

To assess mitochondrial changes, we measured VDAC, a surrogate mitochondrial mass marker. VDAC levels were higher in males, but unaffected by CYB5R3 overexpression (Fig. 2A). For OXPHOS, complex I was unchanged (Fig. 2B), but complex II was decreased in WTM *vs*. WTF (Fig. 2C). Complexes III and IV were elevated in WTM *vs*. WTF, but this difference was diminished by transgenesis (Fig. 2D, E). Complex V increased only in TGF *vs*. WTF, and was higher in TGF than in TGM (Fig. 2F). OXPHOS/VDAC ratios (Supplemental Fig. 1) revealed consistent sex effects: except for complex IV/VDAC (unchanged), the remaining complexes were lower in males, with no effect of transgenesis. Figure 2G depicts western blots used for quantification of VDAC and OXPHOS markers.

### Regulation of hepatic markers of mitochondrial dynamics and biogenesis by sex and CYB5R3 overexpression

**Fig. 3:**
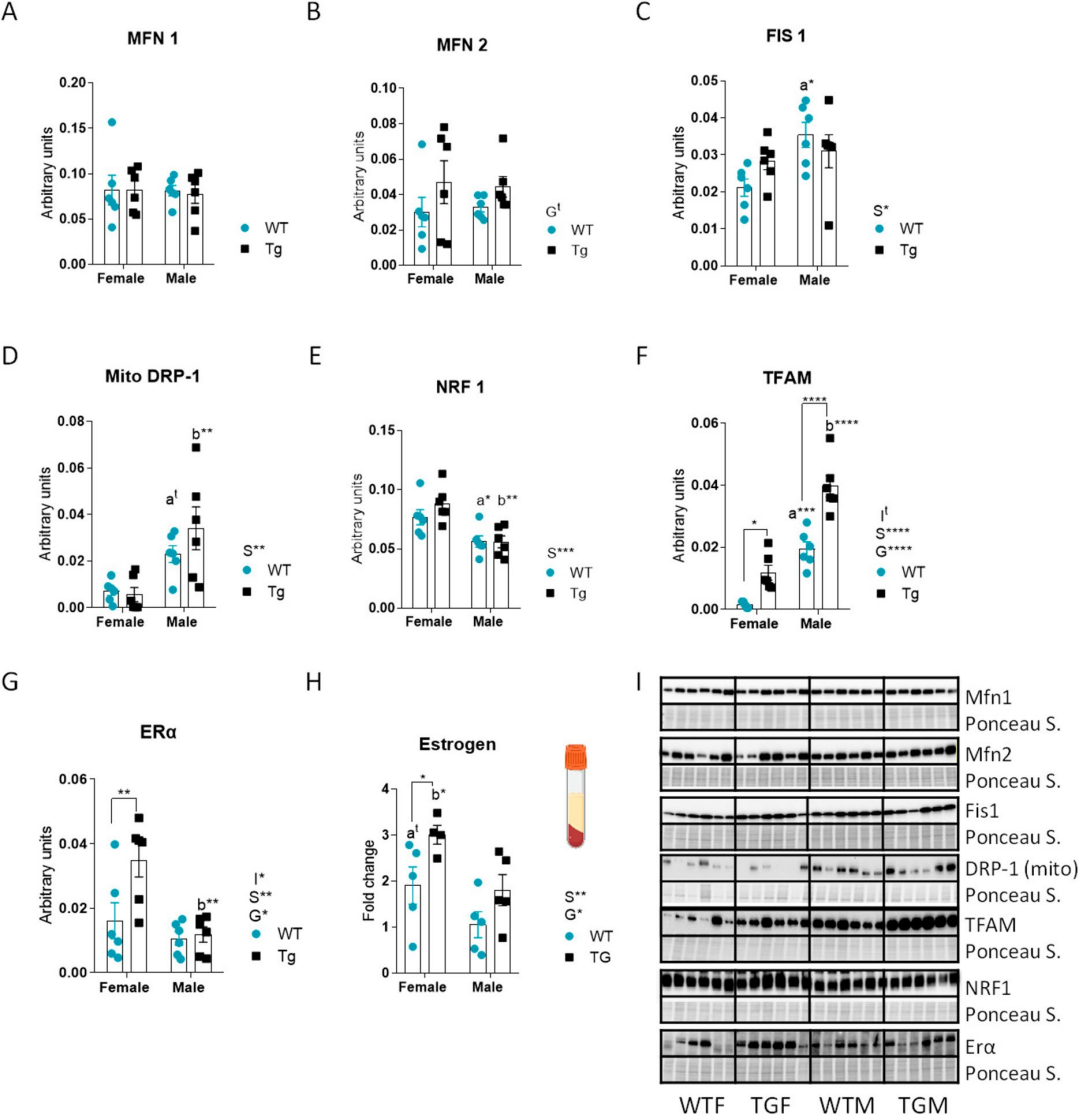
Levels of proteins related to mitochondrial dynamics, biogenesis, estrogen receptor-alpha (ERα) and estrogen: MFN1 (**A**), MFN2 (**B**), FIS1 (**C**), mitochondrial DRP1 (**D**), NRF1 (**E**), TFAM (**F**), ERα (**G**) and estrogen (**H**) in the liver from female and male mice of WT or TG genotypes. Fold change of levels of estrogen in serum from female and male mice of WT or TG genotypes. Western blots used for quantification of protein levels and their corresponding Ponceau S-stained lanes used for normalization of protein loading are shown in panel **I**. Global effects of sex or genotype, as well as the interaction between these factors, were evaluated by two-way ANOVA. Asterisks in the bars denote the level of significance of the differences between genotypes (WT *vs*. TG) for a given sex. Asterisks or “t” (trend) that are accompanied by “a” (for WT) or “b” (for TG) denote significant differences between females and males for a given genotype. Global effects of sex (regardless of genotype) are represented as “S” with asterisks, while overall effects of genotype (regardless of sex) are represented as “G” with asterisks. The interaction “sex x genotype” is indicated as “I” when appropriate. Data are shown as mean ± SEM of 6 animals per group

The mitofusins MFN1 and MFN2 were measured as fusion markers, and FIS1 and DRP1 were measured as fission markers. No statistically significant differences among groups were detected for MFN1, while there was trend towards a main effect of CYB5R3 overexpression to upregulate MFN2 irrespective of sex (see Fig. 3A, B). Regarding the fission markers, FIS1 was found elevated in WTM *vs*. WTF, but no differences were observed when comparing TGM *vs*. TGF (Fig. 3C). We did not evidence any statistically significant change for DRP1 when measured in total homogenate (Supplemental Fig. 2A), but a substantial increase in males *vs*. females was observed in mitochondria-enriched isolated fractions (Fig. 3D). Since most of DRP1 resides in the cytosol under normal cellular conditions but it is translocated to mitochondria during mitochondrial fission [20, 21], we also measured the levels of DRP1 in cytosolic fractions, and found that, as observed for total homogenate, their levels did not differ among groups (Supplemental Fig. 2 B).

We next quantified the levels of NRF1 and TFAM, two key transcription factors regulating mitochondrial biogenesis, and found a different pattern of changes for both markers depending on sex and/or CYB5R3 overexpression. In the case of NRF1, the only change was a decrease in males regardless genotype (Fig. 3E) but, interestingly, TFAM levels were significantly affected by sex (being elevated in males) and by CYB5R3 overexpression both in males and in females (Fig. 3F). While CYB5R3 was not increased in TGM *vs*. WTM (see Fig. 1B), the levels of TFAM were found dramatically elevated in TGM in comparison with the remaining groups (Fig. 3F). Figure 3I depicts western blots used for quantification of different markers with their corresponding Ponceau S-stained lanes used for normalization of protein loading.

We also determined the hepatic levels of the estrogen receptor-alpha (ERα) and found a main effect of sex to increase ERα in females, particularly in those of TG genotype because CYB5R3 overexpression produced a further increase of ERα in females. We also evidenced a main effect of genotype to increase ERα irrespective of sex, but differences were statistically significant only when comparing TGF *vs*. WTF (Fig. 3G). Changes of plasma estrogens with sex and/or transgenesis resembled those of ERα, with main effects of sex (estrogen was more elevated in females) and genotype (estrogen was more elevated in TG mice). Likewise, when comparisons were made among experimental groups, the increase due to CYB5R3 overexpression was statistically significant only in females (Fig. 3H).

### Hepatic markers of fatty acid utilization are regulated by sex and/or CYB5R3 overexpression

**Fig. 4:**
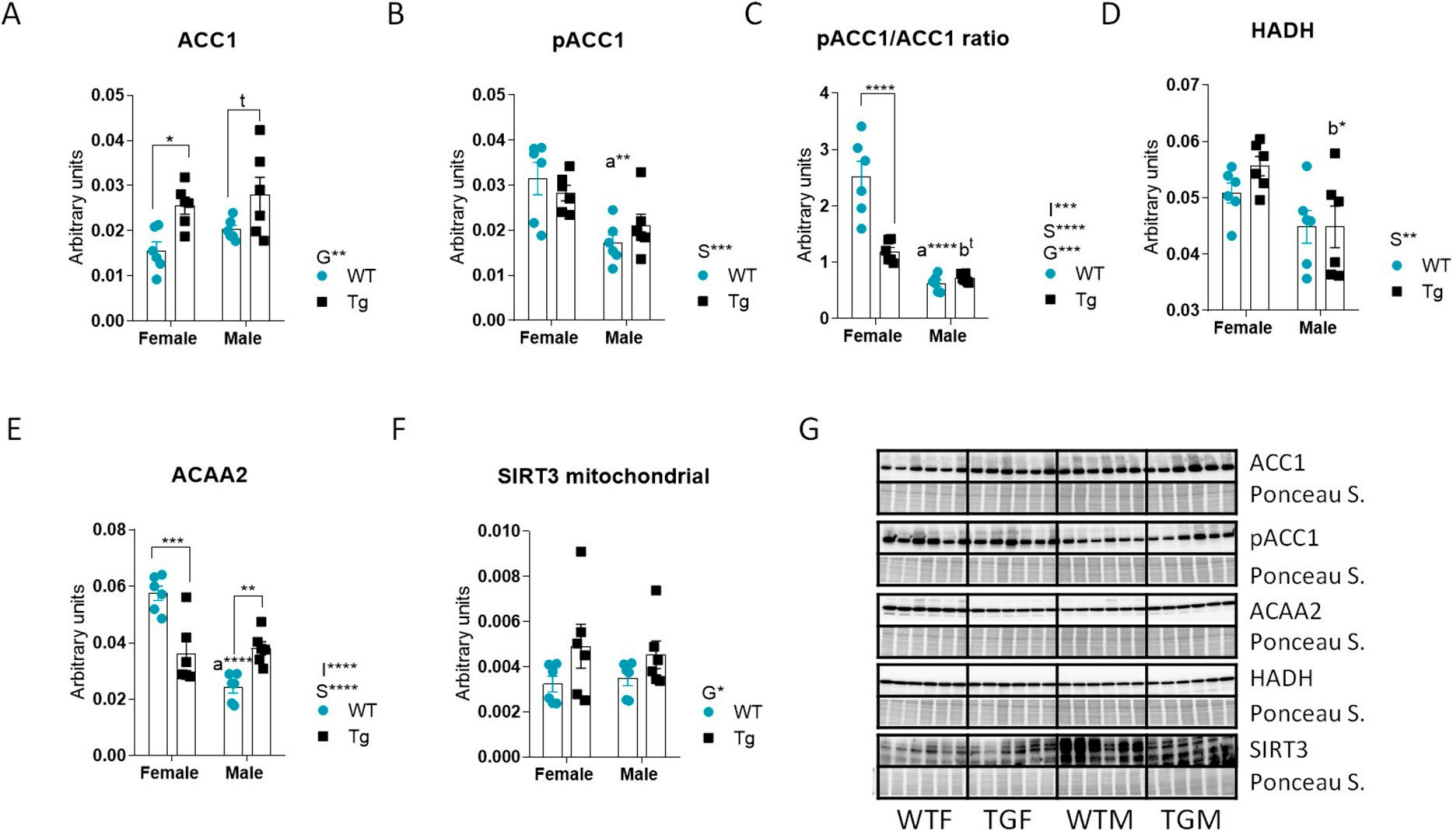
Protein markers related with the regulation of lipid metabolism in the liver from female and male mice of WT or TG genotypes. ACC1 (**A**), pACC1 (**B**), pACC1/ACC1 ratio (**C**), HADH (**D**), ACAA2 (**E**) and SIRT3 (**F**). Western blots used for quantification of protein levels and their corresponding Ponceau S-stained lanes used for normalization of protein loading are shown in panel **G**. Global effects of sex or genotype, as well as the interaction between these factors, were evaluated by two-way ANOVA. Asterisks or “t” (trend) in the bars denote the level of significance of the differences between genotypes (WT *vs*. TG) for a given sex. Asterisks or t that are accompanied by “a” (for WT) or “b” (for TG) denote significant differences between females and males for a given genotype. Global effects of sex (regardless of genotype) are represented as “S” with asterisks, while overall effects of genotype (regardless of sex) are represented as “G” with asterisks. The interaction “sex x genotype” is indicated as “I” when appropriate. Data are shown as mean ± SEM of 6 animals per group

We observed a significant effect of transgenesis increasing total acetyl-CoA carboxylase 1 (ACC1), with statistically significant differences in females (Fig. 4A). Phosphorylated ACC1 (pACC1) exhibited a different pattern of changes, as we evidenced a significant effect of sex leading to decreased pACC1 levels in males, with statistically significant differences between WTM and WTF (Fig. 4B). We then calculated the pACC1/ACC1 ratio and found that both sex and CYB5R3 overexpression had a profound impact on this parameter. Specifically, we observed a main effect of sex to decrease the pACC1/ACC1 ratio in males. This effect was particularly pronounced in WT mice, as this ratio was dramatically reduced by CYB5R3 overexpression in females, thereby narrowing the difference between TGM and TGF (Fig. 4C). The levels of hydroxyacyl-coenzyme A dehydrogenase (HADH) were slightly reduced in males, with statistically significant differences observed between TGM and TGF, but no effect of CYB5R3 overexpression was detected (Fig. 4D). We observed a distinct pattern of changes for acetyl-CoA acyltransferase 2 (ACAA2), with a significant genotype × sex interaction. Specifically, ACAA2 levels were decreased in WTM *vs*. WTF, but not in TGM *vs*. TGF. Additionally, CYB5R3 overexpression decreased ACAA2 levels in females, while it caused an increase in males (Fig. 4E). Mitochondrial (cleaved) SIRT3 exhibited similar changes across experimental groups as those observed for ACC1, with a main effect of genotype resulting in increased mito-SIRT3 levels in TG mice (Fig. 4F). Figure 4G depicts western blots used for quantification of ACC1, p-ACC1, HADH, ACAA2 and SIRT3 markers with their corresponding Ponceau S-stained lanes used for normalization of protein loading.

### CYB5R3 overexpression and sex modulate hepatic autophagy and mitophagy markers

**Fig. 5:**
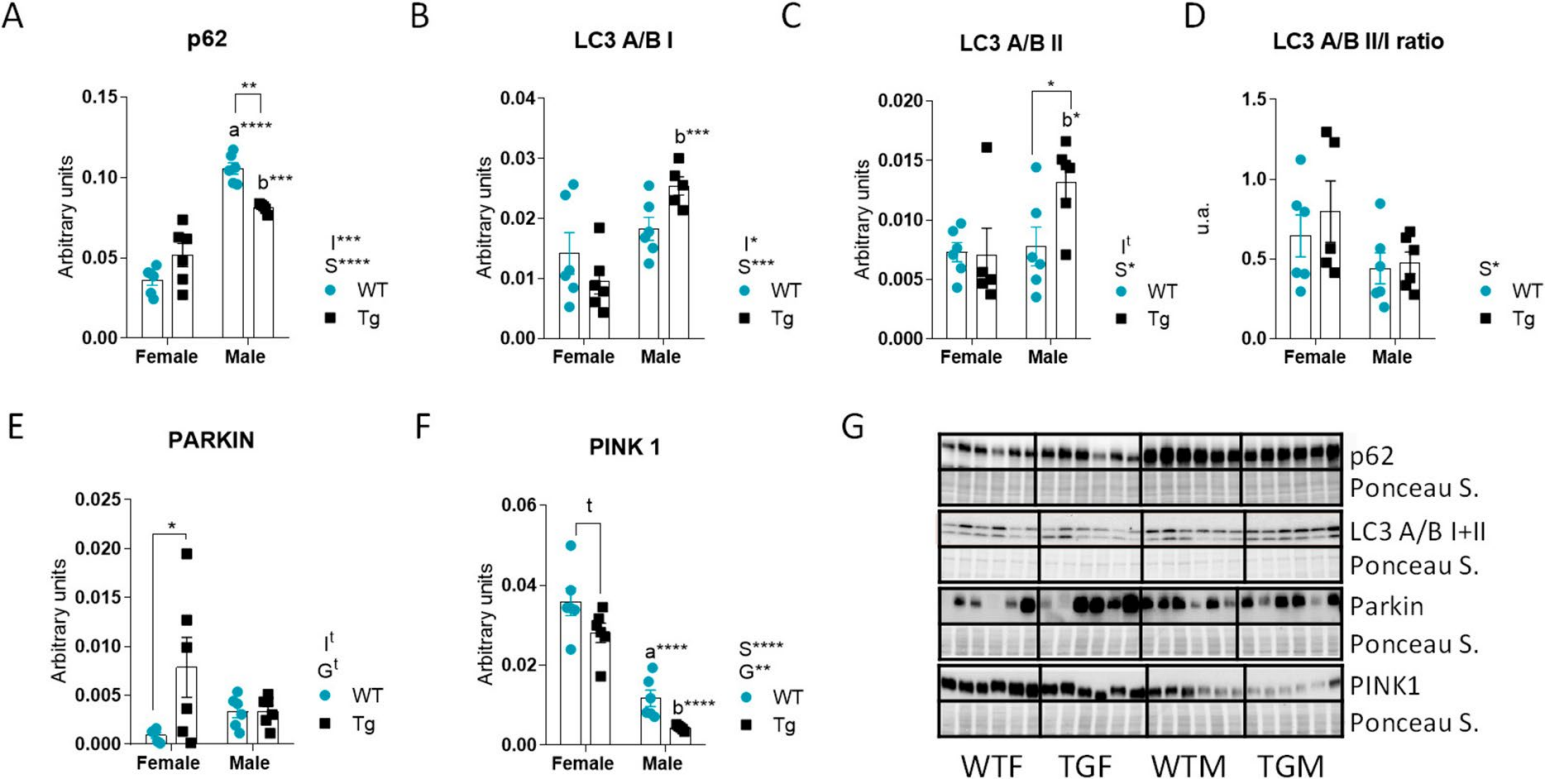
Levels of proteins related to autophagy and mitophagy markers in the liver from female and male mice of WT or TG genotypes. p62 (**A**), LC3 A/B I (**B**), LC3 A/B II (**C**), LC3 A/B II/I ratio (**D**), PARKIN (**E**) and PINK1 (**F**). Western blots used for quantification of protein levels and their corresponding Ponceau S-stained lanes used for normalization of protein loading are shown in panel **G**. Global effects of sex or genotype, as well as the interaction between these factors, were evaluated by two-way ANOVA. Asterisks or “t” (trend) in the bars denote the level of significance of the differences between genotypes (WT *vs*. TG) for a given sex. Asterisks that are accompanied by “a” (for WT) or “b” (for TG) denote significant differences between females and males for a given genotype. Global effects of sex (regardless of genotype) are represented as “S” with asterisks, while overall effects of genotype (regardless of sex) are represented as “G” with asterisks or t. The interaction “sex x genotype” is indicated as “I” when appropriate. Data are shown as mean ± SEM of 6 animals per group

We observed a main effect of sex upregulating p62 in males, with statistically significant differences being observed for both genotypes. CYB5R3 overexpression did not affect p62 levels in females but produced a decrease in males (Fig. 5A). Both LC3A/B I and LC3A/B II were increased in TGM compared with TGF (Fig. 5B, C), and CYB5R3 overexpression also produced a significant increase of LC3A/B II in TGM in comparison with WTM (Fig. 5C). The ratio between LC3A/B II and LC3A/B I was decreased in males but remained unchanged by CYB5R3 overexpression (Fig. 5D). Regarding two of the most relevant proteins involved in the autophagic clearance of mitochondria (mitophagy), PARKIN was significant upregulated by CYB5R3 overexpression in females (Fig. 5E) while a distinct pattern of changes was evidenced for PINK1, as their levels were found significantly elevated in females of both genotypes in comparison with genotype-matched males (Fig. 5F). Figure 5G depicts western blots used for quantification of p62, LC3 A/B I and II, PARKIN and PINK markers with their corresponding Ponceau S-stained lanes used for normalization of protein loading.

### Nutritional status sensors are strongly regulated by CYB5R3 overexpression in a sex-specific way

**Fig. 6:**
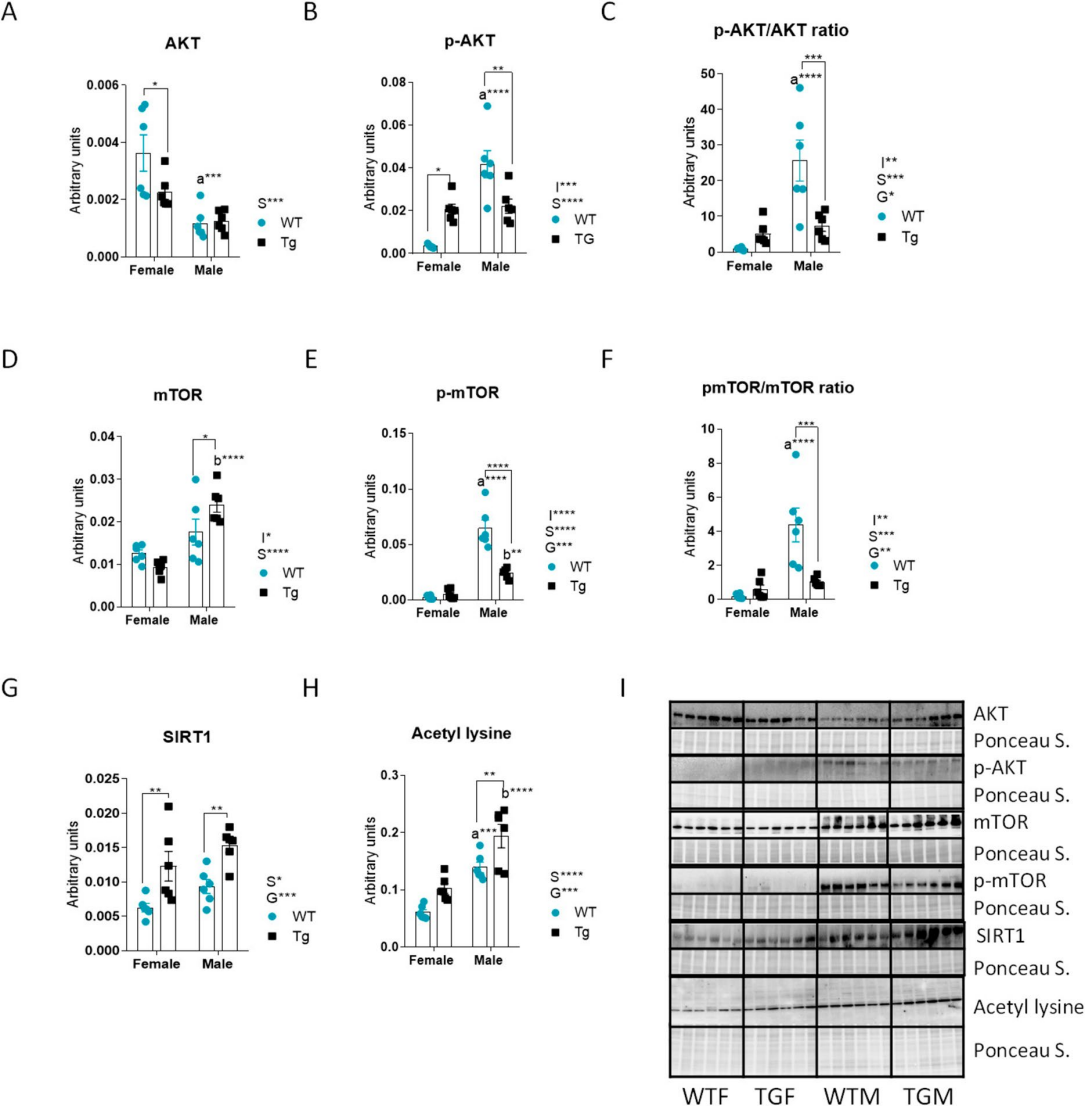
Levels of proteins related to nutritional status sensors and regulation of enzymatic activity and gene expression AKT and mTOR (anabolic/catabolic pathways), SIRT1 and protein pan-acetylation. AKT (**A**), p-AKT (**B**), p-AKT/AKT ratio (**C**), mTOR (**D**), p-mTOR (**E**), p-mTOR/mTOR ratio (**F**), SIRT1 (**G**) and Acetil Lysine (**H**) in the liver from female and male mice. Western blots used for quantification of protein levels and their corresponding Ponceau S-stained lanes used for normalization of protein loading are shown in panel **I**. Global effects of sex or genotype, as well as the interaction between these factors, were evaluated by two-way ANOVA. Asterisks in the bars denote the level of significance of the differences between genotypes (WT *vs*. TG) for a given sex. Asterisks that are accompanied by “a” (for WT) or “b” (for TG) denote significant differences between females and males for a given genotype. Global effects of sex (regardless of genotype) are represented as “S” with asterisks, while overall effects of genotype (regardless of sex) are represented as “G” with asterisks. The interaction “sex x genotype” is indicated as “I” when appropriate. Data are shown as mean ± SEM of 6 animals per group

Total AKT was downregulated in males irrespective of genotype. CYB5R3 overexpression attenuated sex-dependent differences because it produced a further decrease of AKT levels in females (Fig. 6A). Interestingly, the active phosphorylated (Ser 473) form of AKT showed a different pattern of changes that were also dependent on sex and CYB5R3 overexpression with a significant genotype × sex interaction. Minimal levels of phosphor-AKT were found in WTF, whereas its maximal levels were observed in WTM. While CYB5R3 overexpression increased phosphor-AKT in females, it decreased its levels in males, thus blunting the differences between TGF and TGM (Fig. 6B). We also calculated the phosphor-AKT/AKT ratio to evidence a dramatic increase in WTM in comparison with WTF and TGM. The lowest phosphor-AKT/AKT ratio was observed in WTF, and no differences were found when comparing TGM and TGF (Fig. 6C).

Total levels of mTOR were higher in males, and CYB5R3 overexpression further increased its levels in males (TGM *vs*. WTM), but not in females (TGF *vs*. WTF), which also resulted in a significant genotype × sex interaction (Fig. 6D). Of note, the pattern of changes we observed for phosphor-mTOR (Ser2448) and the phosphor-mTOR/mTOR ratio resembled that of phosphor-AKT and phosphor-AKT/AKT ratio, with minimal values being observed in females of both WT and TG genotypes, and a dramatic upregulation in WTM that was attenuated (for phosphor-mTOR) or even abated (for the phosphor-mTOR/mTOR ratio) in TGM (see Fig. 6E, F).

SIRT1 levels were also regulated by both sex (being increased in males independently of genotype) and CYB5R3 overexpression (being increased in TG mice independently of sex). The increase of SIRT1 due to transgenesis was statistically significant both in males (TGM vs. WTM) and in females (TGF vs. WTF) (Fig. 6G). Strikingly, the pan-acetylation of proteins followed a similar pattern to that of the deacetylase because it was increased in males and by CYB5R3 overexpression as well (Fig. 6H). Finally, Fig. 6I shows western blots used for quantification of AKT, p-AKT, mTOR, p-mTOR, SIRT1 and acetyl lysine with their corresponding Ponceau S-stained lanes used for normalization of protein loading.

### The effect of sex and/or CYB5R3 overexpression on ultrastructural features of hepatocytes mitochondria and lipid droplets

**Fig. 7:**
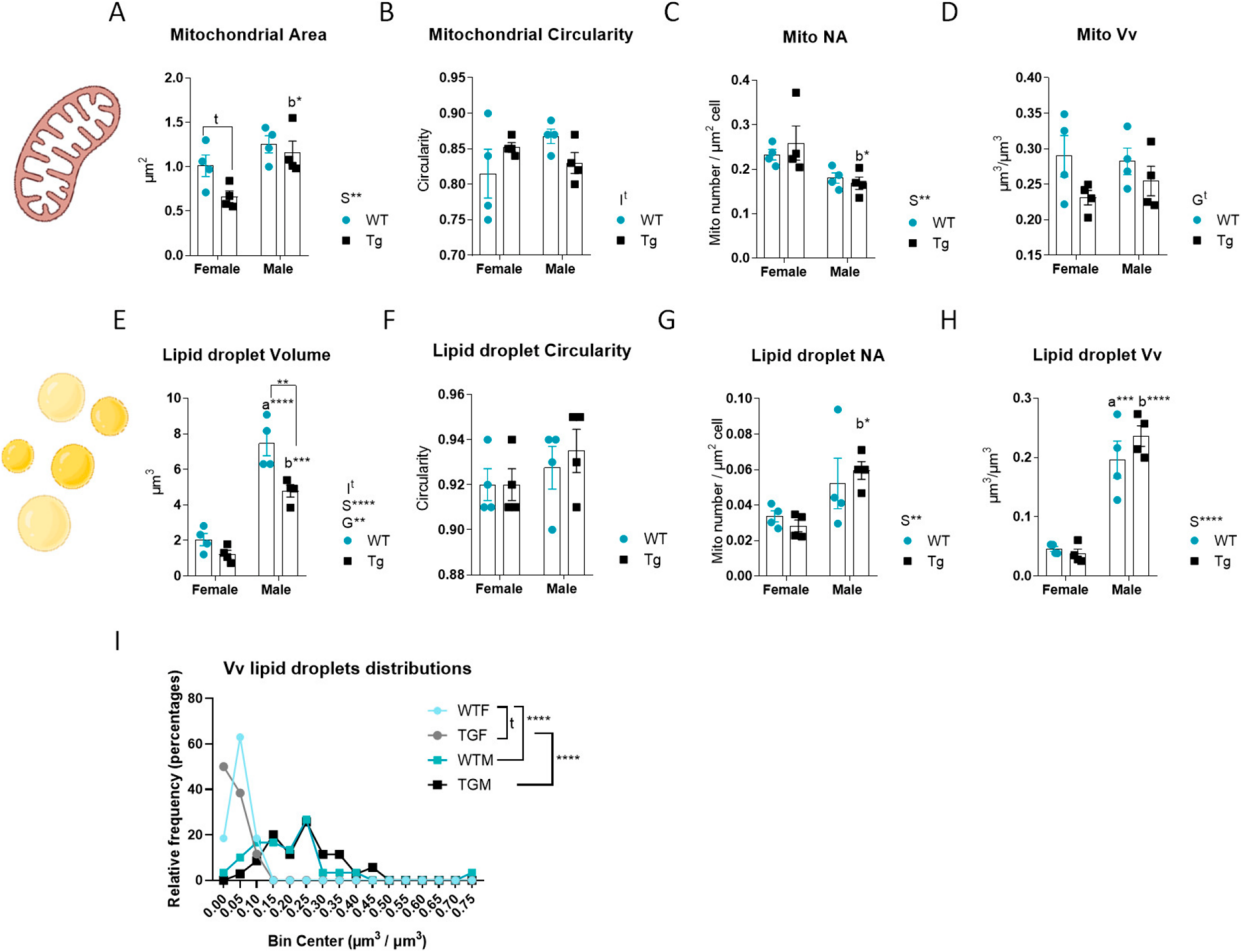
Planimetric and morphometric features as observed in sections from liver left lateral lobe samples obtained from female and male mice of WT or TG genotypes. Planimetric mitochondrial area (**A**), mitochondrial circularity (**B**). Stereological parameters in mitochondrial: Na, a marker of numerical abundance (**C**), Vv, volumetric abundance of the mitochondrial system (**D**). Planimetric lipid droplet volume (**E**), lipid droplet circularity (**F**). Stereological parameters in lipid droplets: Na, a marker of numerical abundance (**G**), Vv, volumetric abundance of the lipid droplets system (**H**), frequency distribution of lipid droplets abundance in hepatocytes (**I**). Global effects of sex or genotype, as well as the interaction between these factors, were evaluated by two-way ANOVA. Asterisks in the bars denote the level of significance of the differences between genotypes (WT *vs*. TG) for a given sex. Asterisks that are accompanied by “a” (for WT) or “b” (for TG) denote significant differences between females and males for a given genotype. Global effects of sex (regardless of genotype) are represented as “S” with asterisks, while overall effects of genotype (regardless of sex) are represented as “G” with asterisks or “t” (trend). The interaction “sex x genotype” is indicated as “I” when appropriate. Data are shown as mean ± SEM of 4–5 animals per group

We finally studied in electron micrographs from hepatocytes if sex and CYB5R3 overexpression produced any alteration in several ultrastructural parameters related with size and shape (by planimetric techniques) and abundance (by stereological techniques) of mitochondria and lipid droplets. In general, CYB5R3 overexpression produced only minor effects on the size and shape of hepatocyte mitochondria. We evidenced a trend towards a decrease in mitochondrial area in TGF *vs*. WTF (Fig. 7A) with no changes in circularity among any of the experimental groups (Fig. 7B). On the other hand, while mitochondrial area was not altered by sex in WT mice, the size was increased in TGM *vs*. TGF (Fig. 7A). Regarding the parameters related with abundance, we found a decrease of Na in males independently of genotype, with statistically significant differences being observed when comparing TGM *vs*. TGF (Fig. 7C). A trend towards decreased Vv in males independently of genotype was also found (Fig. 7D).

**Fig. 8:**
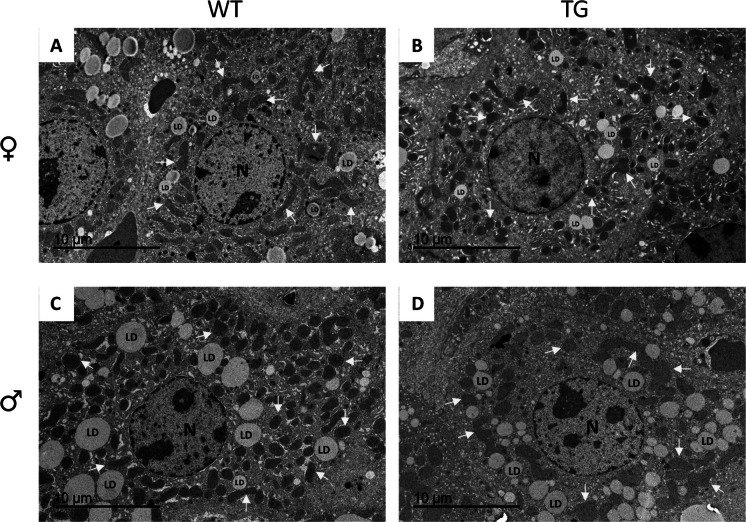
Representative micrographs of hepatocytes obtained from liver left lateral lobe samples from female and male mice of WT and TG genotype, as observed by transmission electron microscopy. **A** WTF. **B** TGF. **C** WTM. **D** TGM. Some examples of the average size of mitochondria (yellow arrows) and lipid droplets (LD) are shown in the pictures. Bars are equal to 10 µm

In contrast to our findings on mitochondrial ultrastructure, the effects of sex and/or CYB5R3 overexpression on lipid droplets were noteworthy. In this case, lipid droplets were significantly larger in males than in females irrespective of genotype, with statistically significant differences for both WT and TG mice. Furthermore, CYB5R3 overexpression produced a decrease in the volume of lipid droplets independently of sex, with statistically significant differences between TGM and WTM (Fig. 7E), without any difference for circularity among experimental groups (Fig. 7F). Parameters related with the abundance of lipid droplets in hepatocytes were also affected significantly by sex. In this case, we evidenced an increase of numerical profile density (Na) in males irrespective of genotype with statistically significant differences being observed for TGM *vs*. TGF (Fig. 7G), as well as an increase of volume density (Vv) in males that was statistically significant for both genotypes (Fig. 7H). We also analysed frequency distribution of lipid droplets abundance in hepatocytes and found strongly dimorphic patterns. In females, most hepatocytes were characterized by a low abundance of lipid droplets, while a small number of hepatocytes from WT but not TG females contained a very high content of lipid droplets. On the contrary, hepatocytes from males were characterized by a broad distribution in terms of lipid droplets abundance, without any difference due to genotype (Fig. 7I). Representative micrographs showing the average size of mitochondria and lipid droplets in hepatocytes from all experimental groups are shown in Fig. 8.

## Discussion

Overexpression of the CYB5R3 gene has emerged as a promising strategy to counteract various metabolic alterations associated with aging, mimicking some of the beneficial effects of CR, the hallmark of anti-aging interventions [10]. However, the impact of CYB5R3 transgenesis is not uniform across sexes because CYB5R3 overexpression primarily enhances carbohydrate metabolism in males [10], whereas it promotes fatty acid oxidation in females [8]. Moreover, biochemical markers adaptations that are associated with CYB5R3 overexpression appear to be more pronounced in either females or males depending on the tissue studied, illustrating the complexity of the CYB5R3-overexpressing transgenic model [8, 11, 13]. In this way, TG females were characterized by increased mitochondrial abundance, upregulation of mitochondrial biogenesis and functional markers in skeletal muscle [11], as well as by the mitigation of age-related changes in mitochondrial size, abundance and ultrastructural characteristics of mitochondria-associated endoplasmic reticulum membranes (MERCS) in kidney distal tubular epithelial cells [12]. In contrast, the upregulation of markers indicative of enhanced mitochondrial function in cardiomyocytes has been predominantly observed in TG males [13]. The existence of sexual dimorphism in the metabolic effects of CYB5R3 overexpression aligns with the broader understanding that both longevity and lifespan extension in response to anti-aging interventions can be strongly influenced by sex in mammals [22].

Our previous research has shown that the accrual of CYB5R3 protein in TG mice is tissue-specific, with significantly higher enrichment in skeletal muscle (particularly in mitochondrial membranes) and heart compared to liver and kidney [8, 10, 23]. However, no study has directly compared TG males and females to their respective WT controls, specifically focusing on the liver. Our results have indicated the existence of post-transcriptional mechanisms regulating CYB5R3 protein levels, especially in males, since the abundance of the endogenous CYB5R3 transcript was higher in WTF that in WTM but the levels of CYB5R3 polypeptide were higher in WTM. Moreover, overexpression of the CYB5R3 gene increased hepatic levels of CYB5R3 transcript both in TGM and in TGF, but the polypeptide was only upregulated in TG females. Data obtained from females align with our previously observed increase of the polypeptide in the liver from 7-month-old TG females [8]. However, the lack of CYB5R3 increase in TG males we show here is in contrast with our previous observations obtained with 7- or 9-month-old TG males [9, 10]. Since the determinations described in this paper were carried out with young (3 months old) mice, it is possible that the consequence of CYB5R3 overexpression in the liver depends on age and is more attenuated in younger animals. Moreover, dietary differences (chow in this study *vs*. AIN93M or AIN93G diets in previous studies [9, 10]) could also influence the results as diet composition impacts the outcomes of pro-longevity interventions [24–26]. Taken together, our results indicate that, in the liver, the accrual of CYB5R3 polypeptide is subjected to posttranscriptional regulation, and may be regulated by factors as sex, age and diet.

We extended our investigation to quantify the levels of CYB5R3 polypeptide in subcellular fractions and confirmed that an increase due to CYB5R3 overexpression was only achieved in females. This approach also enabled us to make a novel and intriguing observation regarding the role of sex in the distribution of endogenous CYB5R3 protein in cytoplasmic organelles. CYB5R3 targeting to mitochondria was dramatically upregulated while targeting to microsomal membranes was significantly downregulated in males compared to females. Notably, CYB5R3 overexpression did not further increase mitochondrial or microsomal targeting in males. Since CYB5R3 is involved in lipid metabolism and many detoxification reactions, the sex-specific targeting of CYB5R3 to different organelles constitutes a level of posttranslational regulation that could contribute to the differential responses of males and females to dietary stresses and toxics [27]. Contrary to the liver, in striated muscles, the abundance of CYB5R3 closely follows that of its transcript and is efficiently overexpressed in TG mice regardless sex. Striated muscles have been thus proposed as good models to investigate cell-autonomous mechanisms of metabolic regulation through CYB5R3 upregulation [8, 11, 23].

Since we had found a sex-specific pattern of CYB5R3 distribution in the liver from WT and TG mice, we wanted to further explore the biochemical signatures that were associated with CYB5R3 overexpression in males and females. Our previous research in cellular systems had documented that CYB5R3 overexpression enhances aerobic respiration [28]. Thus, we focused out attention towards the effect of CYB5R3 overexpression on well-established markers of this process as OXPHOS complex abundance, mitochondrial dynamics and biogenesis, and mitochondrial fatty acid utilization. Our findings have indicated that VDAC, a biochemical surrogate of mitochondrial abundance [29], was increased in males but not affected by CYB5R3 overexpression. We previously showed that this intervention did not affect VDAC levels in skeletal muscle from females [8]. Regarding the markers of electron transporting OXPHOS complexes in the liver, we have found a consistent pattern for CYB5R3 overexpression abating sex-dependent differences (as the decrease of complex II and the increases of complexes III and IV in WTM *vs*. WTF). Only for complex V, we evidenced an increase in TGF *vs*. WTF. The pattern of sex-dependent alterations coincides essentially with that observed in skeletal muscle (except for complex I that remained unchanged among sexes in the liver but was increased in skeletal muscle from WT males *vs*. females) [11]. In the heart, however, all markers of electron transporting OXPHOS complexes, particularly complex IV, were significantly higher in WTM *vs*. WTF [13].

Since we had evidenced significant differences in the abundance of VDAC when comparing males and females, the possibility exists that changes in OXPHOS complex levels among several experimental groups could be accounted by differences of mitochondrial abundance. Thus, we normalized OXPHOS complex abundance to VDAC and found a consistent pattern of changes by sex since, except for complex IV/VDAC that remained unchanged; the normalized levels of the remaining complexes were downregulated in males *vs*. females without any effect of CYB5R3 overexpression. Downregulation of the normalized abundance of OXPHOS complexes in liver mitochondria from males agrees with the general view that respiration capacity is higher in female than in male mitochondria [30]. On the other hand, the lack of alterations in electron transporting complex markers in the liver from females overexpressing CYB5R3 agrees with our previous observations in skeletal muscle [11] and heart [13] from females. In the heart from males, however, overexpression of CYB5R3 produced a generalized decrease of mitochondrial complex markers while maintaining maximal respiration rates, presumably by optimizing the formation and preservation of supercomplexes [13]. Changes in complex I in striated muscles, but not in the liver, may be linked to the well-known differences in substrate utilization between muscle and liver mitochondria. Liver mitochondria primarily rely on complex I-linked substrates, whereas muscle mitochondria predominantly depend on complex II-linked substrates [31].

Mitochondrial homeostasis depends on the biogenesis of new components, coordinated with the characteristic dynamics of fusion and fission. These processes facilitate the mixing of mitochondrial constituents and enable the removal of damaged segments through mitophagy, which is closely associated with fission [32]. Hepatic levels of mitofusins (MFN1 and MFN2) were not altered significantly by either sex or CYB5R3 overexpression, which agrees with our previous study focused on the heart [13]. In skeletal muscle, however, increased levels of MFN1 were observed in WTF *vs*. WTM, although without any change associated to CYB5R3 overexpression [11]. Fission-related markers were strongly regulated by sex in the liver, since FIS1 was found elevated in WTM compared with WTF and, as also found for OXPHOS complexes (see above), the sex-dependent difference was abated by CYB5R3 overexpression. No differences among groups were evidenced for total and cytosolic DRP1, but mitochondria-associated DRP1 was dramatically upregulated in males independently of genotype, supporting a predominance of fission in males *vs*. females. The regulation of fission markers by sex and/or CYB5R3 overexpression in the liver contrasts with the outcome of these factors in striated muscles. In skeletal muscle, FIS1 was found increased in females *vs*. in males, and DRP1 was significantly upregulated by CYB5R3 overexpression in females but not in males [11]. In cardiac muscle, however, FIS1 was significantly upregulated in WTM compared with WTF, and this increase was abated by CYB5R3 overexpression, while the levels of mitochondria-associated DRP1 did not vary among experimental groups [13]. Taken together, the findings described in this and in previous papers are indicative of a stronger effect of CYB5R3 on mitochondrial dynamics in skeletal than in cardiac muscle or liver.

We also encountered striking differences between experimental groups in the markers of mitochondrial biogenesis. The nuclear transcription factor NRF1 was downregulated in males *vs*. in females irrespective of CYB5R3 overexpression. Since NRF1 regulates the expression of many nuclear-encoded genes required for mitochondrial function, including the components of the OXPHOS chain [30], our results are in accordance with the decrease in the normalized abundance of OXPHOS complexes in males *vs*. females (see above). However, whereas TFAM is also a target of NRF1-mediated gene expression [30], this factor followed a different pattern of changes with sex and/or CYB5R3 overexpression. Interestingly, hepatic TFAM was found elevated in males *vs*. in females, and was further upregulated by CYB5R3 overexpression in mice of both sexes. TFAM directly regulates the replication and transcription of mitochondrial DNA (mtDNA) and plays a critical role in maintaining its stability [33]. Its upregulation in males might indicate the necessity to sustain these functions in mitochondria from males that are characterized by a decreased specific abundance of OXPHOS complexes. We previously showed that CYB5R3 overexpression produced a concerted upregulation of both TFAM and NRF1 in skeletal muscles from females but not from males [11], although no such upregulation was observed in the heart, where these markers were significantly elevated in females regardless of genotype [13]. In any case, it is important to consider that, while hepatic TFAM was significantly upregulated in both TGM and TGF, CYB5R3 levels remained unchanged in TGM. This observation supports that non-cell autonomous mechanisms, likely related to inter-organ communication between the liver and other tissues exhibiting efficient overexpression at the polypeptide level (as skeletal muscle and heart), might also contribute to determine hepatic metabolic adaptations.

Steroid sex hormones have been linked to mitochondrial function [34], and we previously suggested that increased estrogenic signalling could influence the outcome of CYB5R3 overexpression at a systemic level [8, 13]. ERα, the most abundant of the estrogen receptors expressed in the liver, promotes mitochondrial biogenesis and enhances mitochondrial function [35–37]. ERα is a critical regulator of hepatic glucose and lipid metabolism which enhances insulin sensitivity, reduces hepatic gluconeogenesis and also supports mitochondrial β-oxidation of fatty acids [38], protecting against metabolic dysfunction-associated steatohepatitis (MASH) by minimizing lipid accumulation, inflammation and fibrosis [39]. Furthermore, ERα signalling is associated with reduced incidence and progression of liver cancer [40]. Strikingly, many of these actions mirror, at least partially, the phenotype described for TG mice [10]. Thus, we were interested in studying how sex and/or CYB5R3 overexpression affected hepatic abundance of ERα and found a main effect of sex to increase its levels in females that is in accordance with previous reports [8]. Of note, ERα upregulation was particularly evident in TGF because CYB5R3 overexpression produced a further increase of ERα levels in this group. We also evidenced a main effect of genotype to increase ERα irrespective of sex, but differences in males did not reach statistical significance. We showed previously that CYB5R3 overexpression also induced a strong increase in the levels of ERα in skeletal muscle from females [8]. In the heart, however, ERα abundance did not vary among experimental groups, indicating that the cardiac phenotype related with CYB5R3 overexpression is not linked to alterations in ERα levels [13]. Interestingly, the pattern of changes of circulating estrogen with sex and/or CYB5R3 overexpression resembled that of hepatic ERα. CYB5R3 is a co-factor of steroid biosynthesis pathways, and both the microsomal and mitochondrial CYB5 isoforms are allosteric effectors that interact with the cytochrome P450c17 oxidoreductase complex to stimulate 17,20-lyase activity in the biosynthetic pathway of sex hormones [41, 42].

Given the reported role of CYB5R3 in regulating mitochondrial fatty acid utilization [8, 10], and the effect of estrogenic signalling that supports mitochondrial fatty acid β-oxidation and reduces lipid accumulation in the liver (see above), we focused on the hepatic levels of key enzymes involved in lipogenesis and lipolysis routes. ACC1, the first and rate-limiting enzyme in de novo fatty acid synthesis, produces malonyl-CoA which inhibits carnitine palmitoyltransferase thereby directing fatty acid metabolism towards lipogenesis rather than β-oxidation, and is strongly inhibited by Ser79 phosphorylation catalysed by AMPK [43, 44]. HADH is a mitochondrial enzyme that participates in the conversion of fatty acids to ketones [43]. Meanwhile, ACAA2 facilitates the final step of mitochondrial fatty acid β-oxidation [43]. Additionally, the sirtuin deacetylase SIRT3 also plays an important regulatory role in substrate utilization, since fatty acid oxidation is impaired in various tissues of SIRT3 knockout mice, likely due to increased acetylation of mitochondrial proteins and/or reduced mitochondrial content [45].

In our previous study focused on liver from 7-month-old females fed a AIN93M diet, we reported no changes in ACC1, pACC1 and HADH, as well as a decrease in ACAA2 in TG females [8]. The decrease in ACAA2, along with the absence of changes in HADH and pACC1, is consistent with the results presented in this paper, although in our previous study we evidenced no changes in ACC1 or the pACC1/ACC1 ratio [8]. The increase in total ACC1 without changes in its phosphorylated form (hence decreasing the pACC1/ACC1 ratio) we show here for TGF *vs*. WTF might be indicative of greater ACC1 activity that leads to carnitine palmitoyltransferase inhibition. This observation, combined with the decrease in ACAA2, is consistent with an increase in hepatic lipogenesis in TGF *vs*. WTF. Hepatic lipids could be then directed to skeletal muscle for energy production via β-oxidation, a metabolic adaptation that has also been observed in RC [46]. This reinforces the idea that phenotypically, TGFs resemble animals subjected to RC [8]. We observed a marked sexual dimorphism in WT mice which is also consistent with increased hepatic lipogenesis in males *vs*. females, i.e., decreases in the pACC1/ACC1 ratio, HADH and ACAA2 in WT males *vs*. females. Although we evidenced an increase of ACAA2 in TGM compared to WTM, overexpression of CYB5R3 had little effect on these markers. No data are available in the literature regarding these metabolic markers in males at different ages.

Autophagy and mitophagy are widely recognized as critical determinants of the rate of aging in animals [15]. As well-established markers of general macroautophagy, we measured p62 and LC3 A/B I and II. We also measured PARKIN and PINK1 as they are specifically involved in mitophagy [47]. The levels of p62 were significantly higher in males than in females. Considering the main effect of sex in reducing the LC3A/B II/I ratio in males, our observations are indicative that the hepatic autophagy is less efficient in males. This agrees with females generally exhibiting higher basal levels of autophagy than males, which helps protect against hepatic lipid accumulation and it is regulated positively by estrogen, thus enhancing lysosomal biogenesis and autophagic flux [48, 49]. Moreover, under fasting conditions, females activate autophagy more effectively, promoting the breakdown of lipid droplets by lipophagy to generate energy. This prevents excessive triglyceride accumulation and protects against steatosis and progression towards metabolic dysfunction-associated steatotic liver disease (MASLD) [49, 50]. Pink1 levels were also much higher in females than in males, which also supports a more active mitophagy in females, as already reported [11]. In addition, the most notable effect regarding PARKIN was its increase in TGF, suggesting that CYB5R3 overexpression might facilitate a further activation of mitophagy in females. Interestingly, in TGM, we evidenced a decrease in p62 along with an increase in both LC3A/B II and I (although resulting in no change in the LC3 ratio) compared to WTM, which could be again indicative of a non-cell autonomous effect of CYB5R3 overexpression on the regulation of autophagic signalling in the liver from TGM. To our best knowledge, no data are available in the literature regarding these autophagy/mitophagy markers in males at different ages.

Nutrient-sensing pathways are also essential for adapting metabolism to nutrient availability. The PI3K-AKT-mTOR pathway is a critical signalling axis for controlling growth, metabolism and longevity, and its dysregulation is involved in diseases like cancer, diabetes and age-related disorders [15, 51]. Thus, we quantified the abundance of total and phosphorylated forms of AKT and mTOR and the results obtained for both metabolic sensors were highly consistent. A notable finding was the significant increases in phosphor-AKT and phosphor-mTOR (as well as in their phosphorylation ratio) in WTM compared to WTF, which is in accordance with the predominance of anabolic pathways in males *vs*. catabolic pathways in females. Of note, these increases were attenuated in TGM compared to WTM, which is consistent with the previously observed decrease in p62 and increases in LC3A/B I and II.

Regarding protein pan-Lys acetylation, we observed an increase in males *vs*. females regardless genotype, as well as an increase in TG mice regardless sex. Lys-acetylation is related to the balance between acetylases and deacetylases (sirtuins), as well as the levels of acetyl-CoA [52]. Strikingly, if we compare these data with the levels of the measured enzymes that generate (ACAA2) or consume (dephosphorylated ACC-1) acetyl-CoA, we found an inverse relationship, as increased consumption and reduced generation would be expected in males. However, the notion that Lys acetylation causes broad-ranging damage to mitochondrial quality and performance has been recently challenged after the demonstration that the functional phenotype of hyperacetylated mitochondria in a double-knockout mouse model with a genetic ablation of both carnitine acetyltransferase and SIRT3 is largely normal. This indicates that redox balance and carbon flux are modulated by the acetyl-Lys turnover rather than by acetyl-Lys stoichiometry [53]. Interestingly, both SIRT1 and SIRT3 were increased in males independently of genotype and in TG mice independently of sex, indicating higher turnover of acetylation-deacetylation, particularly in TGM. We found previously a similar pattern in skeletal muscle, combining the increase of SIRT3 with higher levels of acetylated proteins in TGF *vs*. WTF [11]. However, whereas the protein pan-Lys acetylation also increased in skeletal muscle from TGM *vs*. TGF, SIRT3 levels were decreased [11]. For SIRT1, the pattern of changes with sex and/or genotype we have found in the liver is also different to that of skeletal muscle, since SIRT1 levels were elevated in males of both genotypes but no effect of CYB5R3 overexpression was observed [11].

Adaptations in mitochondrial structure, including changes in size and shape, are strongly linked to processes such as aging, exercise and anti-aging interventions [54]. Our findings have revealed that sex imposes a more pronounced impact on mitochondrial morphology than CYB5R3 overexpression. Mitochondrial circularity remained unchanged across all experimental groups. In males, mitochondrial size was increased regardless of genotype, while numerical abundance (Na) was reduced, with statistically significant differences observed in TG mice for both parameters. Consequently, the volume density (Vv) showed no variation between the groups. Again, these changes contrast with the effects of sex and/or CYB5R3 overexpression in striated muscles and kidney. In the heart, CYB5R3 overexpression did not affect the planimetric parameters of the two types of mitochondria in females but increased the size of both subsarcolemmal mitochondria (SSM) and intermyofibrillar mitochondria (IMFM) in males without changes in circularity and in the stereological parameters related with abundance (Na and Vv) [13]. In skeletal muscle, no changes were found for SSM in both sexes, but IMFM were larger in TGF and smaller in TGM. Moreover, circularity of IMFM was decreased and Na increased in TGF [11]. In epithelial cells from kidney distal tubules, CYB5R3 overexpression mitigated age-related changes in mitochondrial size and abundance, producing its effects mostly in females as well [12].

For lipid droplets, we evidenced that their ultrastructural features in hepatocytes were strongly dependent on sex, as both size and abundance were dramatically increased in males *vs*. females irrespective of genotype, without any difference in circularity. In accordance, female mice exhibit lower hepatic fat accumulation and a decreased propensity for inflammation and fibrosis and are less susceptible to MASLD and hepatic cancer compared to males, which is related with the protective effect of estrogen [49]. Interestingly, while CYB5R3 overexpression did not affect the ultrastructural features of lipid droplets in females, it led to a decrease of their size in males, although no differences in Vv were encountered when comparing TGM *vs*. WTM because their relative abundance (Na) was also increased in TGM. The size of lipid droplets in hepatocytes has been proposed as a marker of lipid metabolic status and a determinant of liver health, since small lipid droplets have been associated with metabolic flexibility, whereas larger lipid droplets may be indicative of pathological lipid accumulation, disrupting liver physiology and contributing to systemic metabolic disorders. Maintaining a balance in lipid droplets size through lifestyle and/or therapeutic interventions is thus critical for liver and overall metabolic health [55]. According to these antecedents, ultrastructural differences we highlight here for the first time might be indicative of an optimization of lipid metabolism of hepatocytes induced by CYB5R3-overexpression in males that could have important implications in preventing metabolic dysfunction that is associated with aging and many pathological states [39, 50]. The changes we have shown for ACAA2, p62, LC3 A/B I and I, and nutritional state sensors (phosphor-AKT, phosphor-mTOR, SIRT1 and protein pan-acetylation) in TGM *vs*. WTM are also consistent with this idea. Indeed, we previously reported that, compared with WTM, TGM showed greater sensitivity to insulin and improved glucose homeostasis when fed a high-fat diet, exhibiting less inflammation and oxidative damage, as well as greater protection against induced hepatic cancer [10].

On the other hand, it has been also reported that the accumulation of lipid droplets within hepatocytes is heterogeneous, and follows a skewed distribution, which is related with fluctuations in the biochemical pathways that control lipolysis, fatty acid oxidation and protein synthesis, contributing to cell-to-cell heterogeneity. This generates a subpopulation of cells that effectively accumulates more lipid droplets and produces more ROS which, interestingly, reduces the risk of lipotoxicity to the general population without impairing overall lipid homeostasis, since high-lipid cells can supply lipids to the other cells [56]. We confirmed the existence of a small population of hepatocytes containing a very high abundance of lipid droplets in WT but not TG females while, in males, we evidenced a broad distribution of lipid droplets among the hepatocyte population without any effect of CYB5R3 overexpression.

In conclusion, our findings reveal that the hepatic abundance of CYB5R3 is sexually dimorphic and is regulated by post-transcriptional and post-translational mechanisms that determine not only the abundance of CYB5R3 protein but also its distribution among cytoplasmic organelles. CYB5R3 gene overexpression increased hepatic CYB5R3 polypeptide in females but not in males, resulting in distinct biochemical profiles that differ by sex. Notably, despite the absence of increased hepatic CYB5R3 in TGM, significant alterations were observed in key markers of mitochondrial biogenesis, autophagy and anabolic and catabolic pathways, and the size of hepatocyte lipid droplets was also decreased. These results indicate that non-cell-autonomous mechanisms, potentially involving inter-organ communication, also play a crucial role in shaping the sex-specific and tissue-specific effects of CYB5R3 overexpression in mice.

## Supplementary Information

Below is the link to the electronic supplementary material.

ESM 1 (PNG 109 KB)

Supplementary file1 (TIF 542 KB)

ESM 2 (PNG 103 KB)

Supplementary file2 (TIF 667 KB)

Supplementary file3 (DOCX 22 KB)

## Data Availability

The data that support the findings of this study are available from the corresponding author upon reasonable request.

## Abbreviations

CLAP: Chymostatin, leupeptin, antipain and pepstatin A
CoQ: Coenzyme Q
CYB5R3: Cytochrome *b*_5_ reductase 3
DMSO: Dimethyl sulfoxide
DTT: Dithiothreitol
EDTA: Ethylenediaminetetraacetic acid
EGTA: Ethylene glycol-bis(β-aminoethyl ether)-*N*,*N*,*N*′,*N*′-tetraacetic acid
ERα: Estrogen receptor α
IMFM: Intermyofibrillar mitochondria
LD: Lipid droplet
Na: Number of figures per area
OXPHOS: Oxidative phosphorylation
PMSF: Phenylmethylsulphonyl fluoride
RIPA: Radioimmunoprecipitation assay
SAEX: Service of Experimentation Animals of the University of Córdoba
sGC: Soluble guanylate cyclase
SSM: Subsarcolemmal mitochondria
TG: Transgenic mice overexpressing CYB5R3
TGF: Transgenic females
TGM: Transgenic males
Vv: Volume density
WTF: Wild-type females
WTM: Wild-type males

## Declarations

All animals were cared for in accordance with the University of Córdoba policy for animal welfare, which complies current European, Spanish and Andalusian regulations and is in accordance with the Guide for the Care and Use of Laboratory Animals published by the US National Institutes of Health and the 1964 Declaration of Helsinki and its later amendments. This study was approved by the bioethics committee of the University of Córdoba and authorized by the Consejería de Agricultura, Pesca y Desarrollo Rural, Junta de Andalucía (authorization code: 8/03/2019/029).
