# Supplementary figures and images for: Adaptations of mitochondrial, autophagy and nutrient sensing pathways in the liver from long-lived mice overexpressing CYB5R3 are sex-dependent and involve inter-organ responses
Source: GeroScience. 2025 Jun 28;48(1):1499–521. doi: 10.1007/s11357-025-01761-z (PMC12972396; doi:10.1007/s11357-025-01761-z)

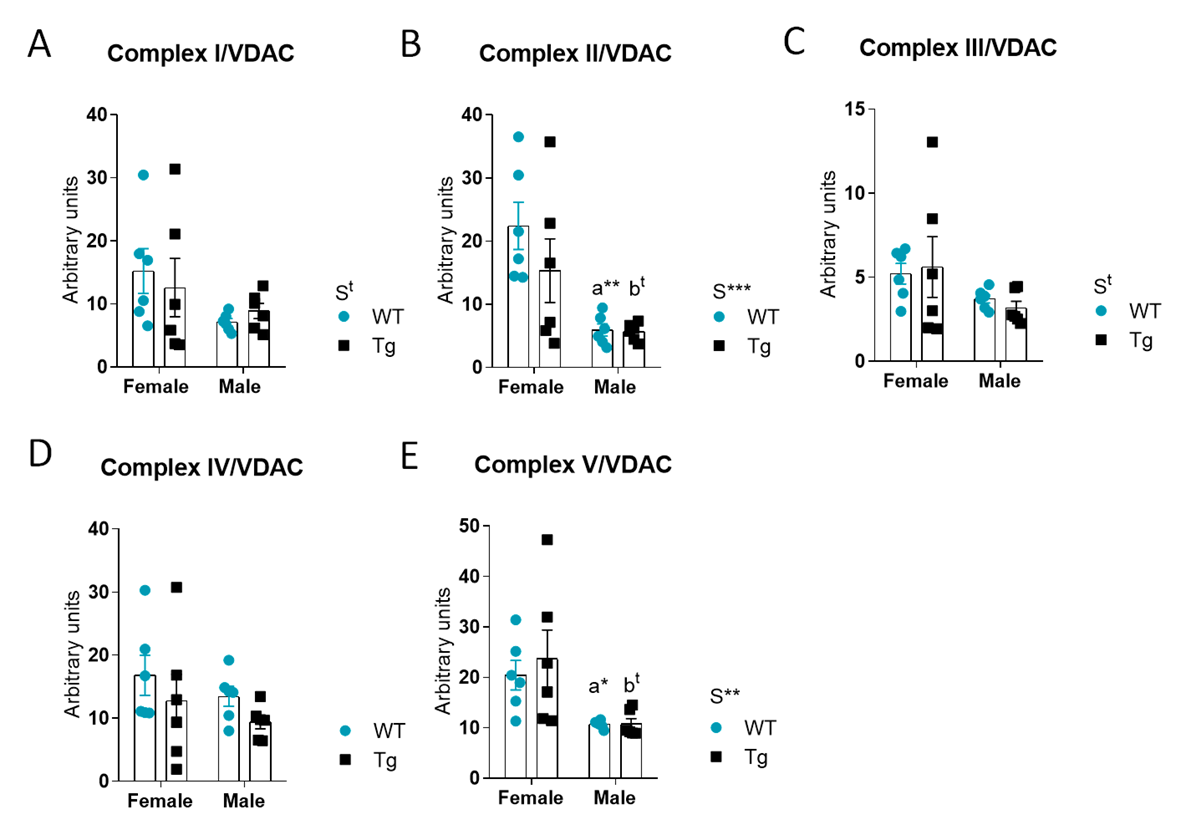

Supplement: Supplementary file 1 — (PNG 109 KB) [file 11357_2025_1761_Fig9_ESM.png]

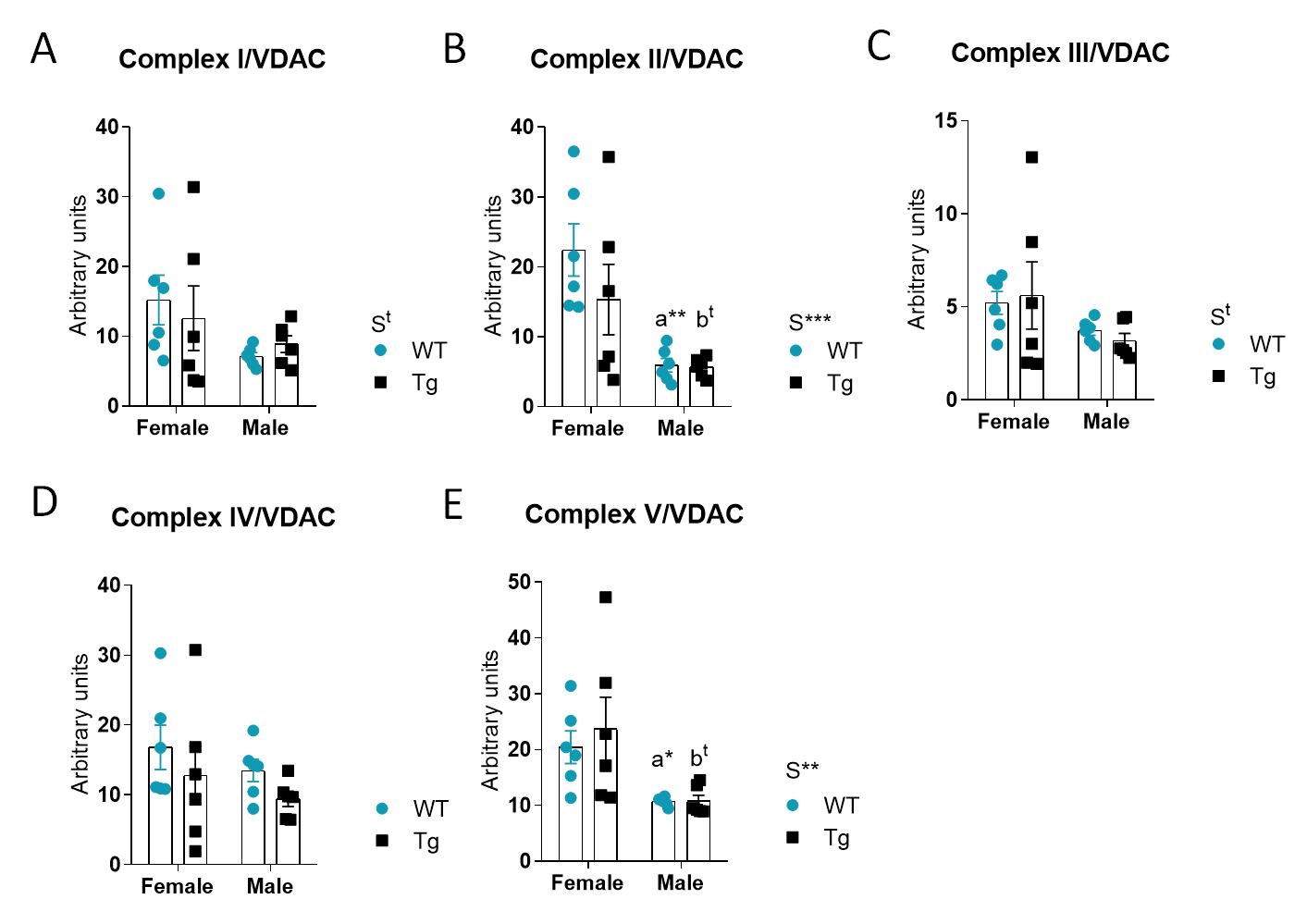

Supplement: Supplementary file 2 — Supplementary file1 (TIF 542 KB) [file 11357_2025_1761_MOESM1_ESM.tif]

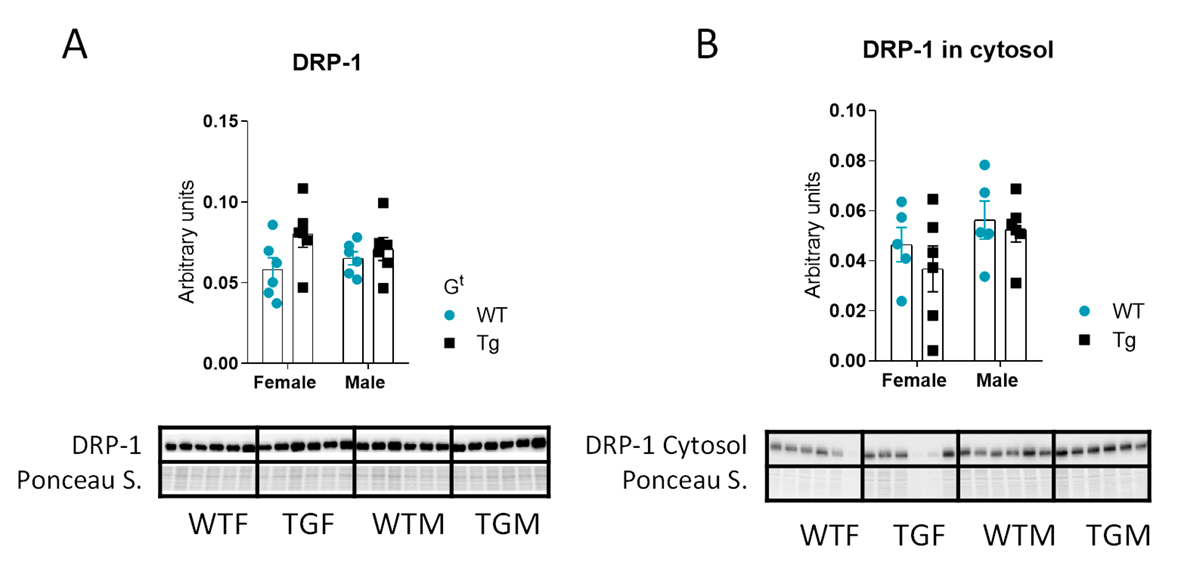

Supplement: Supplementary file 3 — (PNG 103 KB) [file 11357_2025_1761_Fig10_ESM.png]

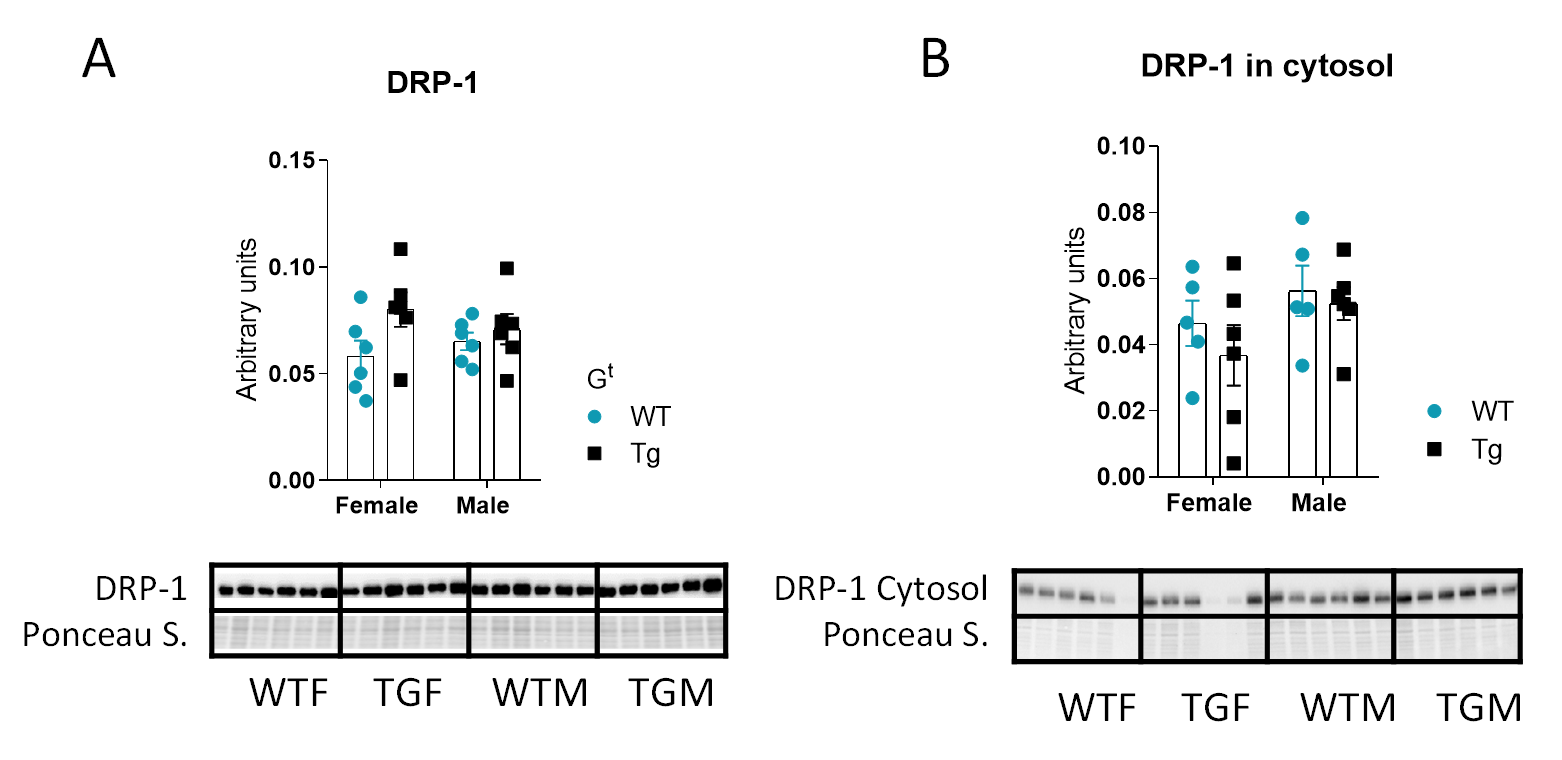

Supplement: Supplementary file 4 — Supplementary file2 (TIF 667 KB) [file 11357_2025_1761_MOESM2_ESM.tif]
