# Supplementary material for: Adaptations of mitochondrial, autophagy and nutrient sensing pathways in the liver from long-lived mice overexpressing CYB5R3 are sex-dependent and involve inter-organ responses
Source: GeroScience. 2025 Jun 28;48(1):1499–521. doi: 10.1007/s11357-025-01761-z (PMC12972396; doi:10.1007/s11357-025-01761-z)
Supplement: Supplementary file 5 — Supplementary file3 (DOCX 22 KB) [file 11357_2025_1761_MOESM3_ESM.docx]

**SUPPLEMENTARY INFORMATION**

**Supplemental Methods**

**Generation of transgenic mice overexpressing CYB5R3.**

The rat CYB5R3 gene was cloned in pRC/CMV-rDTD plasmid between cytomegalovirus immediate-early promoter and SV40 poly-adenylation sequences [1]. After digesting the vector with SwaI and NruI restriction enzymes, the cleaved construct was microinjected into fertilized C57BL/6J eggs at the University of Michigan Transgenic Animal Model Core Facility (<http://www.med.umich.edu/tamc/>). Stable incorporation of the construct was validated as previously reported [2]. Surviving eggs were transferred to pseudopregnant B6D2F1 female mice. Presence of the transgene was detected by PCR genotyping with DNA obtained from tail tissue, using the primers CACCAAAATCAACGGGACTT (forward) and AGACCGGGGAGAGTACCACT (reverse). In each PCR reaction we also carried out the simultaneous amplification of the IL2 gene using the primers CTAGGCCACAGAATTGAAAGATCT (forward) and GTAGGTGGAAATTCTAGCATCATCC (reverse) as internal control. To establish our cohorts, we crossed transgenic males WT females of the same genetic background (C57BL/6J) purchased from Charles River (Barcelona, Spain), and males and females of both genotypes (n=5-7 per group) were then selected from the resulting offsprings.

**Tissue processing for electron microscopy**

Small pieces (about 1 mm^3^) from the left lateral lobe of the livers were obtained and quickly fixed in 2.5 % glutaraldehyde in 0.1M sodium cacodylate buffer pH 7.2 for 12-24 h and then washed in buffer and post fixed in 1% osmium tetroxide for 1 h at 4 °C in the same buffer. The samples were then dehydrated in an ascending series of ethanols, transferred to propylene oxide, and infiltrated in EMbed 812 resin (Electron Microscopy Sciences, PA, USA) using the sequence: propylene oxide:resin 2:1, 1:1, and 1:2 throughout 24 h (8 h each), and pure resin for 24 h. After infiltration, tissue pieces were transferred to silicon moulds containing fresh resin. The blocks were finally cured for 48 h at 65 °C to allow for polymerization. Blocks were trimmed from excess resin and sectioned in an Ultracut Reicher ultramicrotome. Thin sections (40-70 nm thick) were mounted on nickel grids and stained in aqueous 2% uranyl acetate and lead citrate ready-to-use (Electron Microscopy Sciences, PA, USA).

**Supplemental Figure 1.** Levels of OXPHOS complexes relative to VDAC content in liver from females and males of WT or TG genotypes. Panels A to E sequentially show the results for each mitochondrial complex. Asterisks in the bars denote the level of significance of the differences between genotypes (WT vs. TG) for a given sex. Asterisks or “t” (trend) that are accompanied by “a” (for WT) or “b” (for TG) denote significant differences between females and males for a given genotype. Global effects of sex (regardless of genotype) are represented as “S” with asterisks or “t”, while overall effects of genotype (regardless of sex) are represented as “G” with asterisks or “t”. The interaction “sex x genotype” is indicated as “I” when appropriate. Data are shown as mean ± SEM of 6 animals per group.

**Supplemental Figure 2.** Levels of DRP1 in whole homogenates (A) and in fractions of cytosol-enriched (B) in liver from females and males of WT or TG genotypes. Western blots used for quantification of protein levels and their corresponding Ponceau S-stained lanes used for normalization of protein loading are shown below their corresponding graph. Global effects of sex or genotype, as well as the interaction between these factors, were evaluated by two-way ANOVA. Global effects of genotype (regardless of sex) are represented as “G” with “t” (trend). Data are shown as mean ± SEM of 6 animals per group.

**References**

1. Belcourt, M.F., et al., *The intracellular location of NADH : cytochrome b(5) reductase modulates the cytotoxicity of the mitomycins to Chinese hamster ovary cells.* Journal of Biological Chemistry, 1998. **273**(15): p. 8875-8881.

2. Martin-Montalvo, A., et al., *Cytochrome b(5) reductase and the control of lipid metabolism and healthspan.* Npj Aging and Mechanisms of Disease, 2016. **2**: p. 12.
